# Geno-guided targeting of ferroptosis: a multi-layered network to overcome radioresistance in lung cancer

**DOI:** 10.3389/fcell.2026.1886712

**Published:** 2026-09-24

**Authors:** Shifang Sang, Guangchao Lv, Shiyun Feng, Hui Zhao, Yulong Ren, Dianlong Jin, Kaizhong Wang

**Affiliations:** 1 Department of Thoracic Surgery, The First Hospital of Jilin University, Changchun, Jilin, China; 2 Department of Histology and Embryology, College of Basic Medical Sciences, Jilin University, Changchun, Jilin, China

**Keywords:** antioxidants, cuproptosis, ferroptosis, lung cancer, radiotherapy

## Abstract

Lung cancer is one of the most prevalent and lethal malignancies worldwide, and radiotherapy serves as a standard therapeutic modality for the management of advanced or inoperable non-small cell lung cancer. However, radioresistance remains a major obstacle that compromises therapeutic efficacy and drives tumor recurrence. Ferroptosis—an iron-dependent, lipid peroxidation-governed form of regulated cell death—substantially contributes to radiation-mediated tumor cell elimination. This review establishes a multi-layered regulatory network through which ferroptosis mediates radioresistance in lung cancer, spanning genetic mutations, epigenetic and RNA-based regulation, protein post-translational modifications, and lipid metabolism reprogramming. Unlike existing reviews, this paper stratifies ferroptotic vulnerability and radiosensitivity according to common driver genotypes—including TP53, LKB1and KEAP1—and proposes a genotype-guided paradigm for precise radiosensitization. By integrating these molecular layers, review provides a supplementary theoretical framework for optimizing radiotherapy in lung cancer and highlights how targeting ferroptosis can overcome resistance in a mutation-specific manner. Furthermore, the crosstalk between ferroptosis and cuproptosis represents a promising combinatorial therapeutic avenue that warrants further preclinical and clinical investigation.

## 1 Introduction

Lung cancer ranks among the most prevalent malignancies worldwide and remains one of the leading causes of cancer-related deaths, with non-small cell lung cancer (NSCLC) accounting for the majority of cases. Radiotherapy serves as the first-line primary therapeutic strategy for advanced lung cancer and inoperable NSCLC patients. With the continuous advancement and optimization of imaging and radiotherapy technologies such as stereotactic body radiation therapy (SBRT) and four-dimensional computed tomography (4DCT), the precision of radiotherapy has been markedly improved. This progress substantially alleviates radiotherapy-related adverse events, while elevating the local control rate and clinical efficacy of malignant tumors. Currently, conventional clinical practice combines radiotherapy with chemotherapy, targeted therapy and immunotherapy to prolong the overall survival of lung cancer patients. Growing efforts have been devoted to exploring the combination of radiotherapy and immune checkpoint inhibitors (ICIs) to amplify anti-tumor therapeutic effects. Meanwhile, the combination of radiotherapy with emerging therapeutic agents has exhibited considerable potential in tumor radiosensitization. Nevertheless, radiotherapy still has inherent limitations, including radioresistance, pneumotoxicity, cardiotoxicity, and even the risk of secondary malignancy. Among these challenges, radioresistance drives tumor cell tolerance to radiation and lung cancer recurrence, representing a major bottleneck hindering clinical treatment outcomes (Cammareri et al., 2026). Clinically, some patients develop persistent primary lesions or distant metastases after receiving standard-dose radiotherapy, suggesting that tumor cells have intrinsic or acquired resistance mechanisms to ionizing radiation (IR) (Dağkazanlı et al., 2026). Current studies have demonstrated that radioresistance involves multiple mechanisms, including enhanced DNA damage repair capacity, dysregulated cell cycle progression, stem cell properties, apoptosis evasion, and tumor microenvironment remodeling (Li L. et al., 2024; Aishajiang et al., 2026). In particular, a subset of radioresistant cells exhibit increased tolerance to ferroptosis, suggesting that this pathway may be partially involved in the regulation of radiation response (Lin et al., 2024).

Ferroptosis, a heavily investigated regulated cell death modality over the past decade, is governed by an intricate regulatory network built upon three core modules, including disrupted iron homeostasis, massive buildup of lipid peroxides, and failure of antioxidant defense systems. Dysregulation of any single module is capable of reshaping the susceptibility of tumor cells toward ferroptosis. In addition, cellular adaptive remodeling and pharmacological interventions exert prominent influences on ferroptotic sensitivity, which creates opportunities for combinatorial therapeutic regimens. (Xing N. et al., 2023; Lin et al., 2024; Li Q. et al., 2025).

Radiation eliminates tumor cells partly by triggering ferroptosis. Radiotherapy can directly initiate ferroptosis within tumor cells, a process driven by reactive oxygen species (ROS) burst and disrupted iron metabolism. However, lung cancer cells can evade ferroptosis through a variety of mechanisms, thereby attenuating the efficacy of radiotherapy. Multiple dimensions, from DNA mutations and RNA regulation to protein modifications, as well as cellular metabolism, partially alter sensitivity to ferroptosis. For instance, KEAP1 mutation leads to compensatory activation of Nrf2 thereby mediating radioresistance (Sun et al., 2016; Patel et al., 2025). SPARC protein mediates radioresistance by inhibiting ferroptosis in a NSCLC radioresistance model, while engineered radiosensitizer can restore the oxidative state and enhance radiosensitivity by degradation of SPARC and suppressing the adaptive upregulation of GPX4/SLC7A11 (Aishajiang et al., 2026). Therefore, available studies support the hypothesize that lung cancer cells develop radioresistance via ferroptosis evasion, mainly relying on intrinsic defensive systems. Targeting certain regulatory pathways may thus enhance ferroptosis in lung cancer cells and reverse radiation resistance, such as pharmacologic interventions (e.g., RSL3, Cabozantinib, and Auranofin) and nanodrug delivery systems (DHA-N@M) (Chen Y. et al., 2025; Li H. et al., 2025; Wang C. Y. et al., 2025). It should be noted that during ferroptosis evasion in the context of radiotherapy, the antioxidant defense systems serve as the primary defense line of cells against ferroptosis and oxidative stress. The adaptive upregulation of antioxidant defense systems constitutes a significant driver of radioresistance. Therefore, weakening the cellular antioxidant defense system is the target of most current sensitization strategies.

Xing mapped the core ferroptosis machinery and its druggable nodes in lung cancer with an emphasis on pharmacological intervention (Xing N. et al., 2023). Guo provided a broad account of ferroptosis across pulmonary diseases and lung cancer, including its crosstalk with other regulated cell death modalities. In addition, radiation resistance was only considered as a secondary branch, ionizing radiation (IR) was not considered as the core perturbation factor, and the specific antioxidant compensatory network after radiotherapy was not analyzed. (Guo D. et al., 2025). Furthermore, Chen surveyed ferroptosis-based radiosensitization across tumor types, briefly stating TP53 and KEAP1 as modulators of ferroptosis and advocating patient selection for ferroptosis-inducing therapy in general terms (Chen et al., 2026). Three previous reviews only listed sensitizing drugs without hierarchical medication logic. This review introduces genomic driver mutations, RNA/m6A/ceRNA epigenetic regulation, protein post-translational modifications (ubiquitination/acetylation), and lipid metabolism reprogramming, and constructs an exclusive multi-level interaction network for radiation stress, which complements m6A, deubiquitinases, lipid droplet metabolism, and other new radioresistance mechanisms in recent years. There is no systematic series of four-layer pathway interactions in the existing reviews. More importantly, this study used ionizing radiation as the core stimulus, and the four major driver genotypes of NSCLC (single mutation/compound co-mutation) were used as the stratified scale. For each mutation, the defense axis of rate-limiting ferroptosis after radiotherapy was defined, and the targeted drugs and drug efficacy detection markers were matched. In addition, the ferroptosis/copperptosis metabolic coupling pathway is added, and a genotype-stratified four-step clinical transformation model is proposed to fill in the logic of individualized radiotherapy sensitization that is missing in the existing reviews.

## 2 Cellular homeostatic mechanisms of ferroptosis

Ferroptosis is a special mode of regulated cell death caused by iron-driven lipid peroxidation and plasma membrane rupture, which is significantly different from other cell death modes such as apoptosis and autophagy. Its hallmark events consist of iron overload, lipid peroxidation and antioxidant system collapse, accompanied by characteristic mitochondrial morphological and metabolic remodeling (Zeng et al., 2023). Since the concept of ferroptosis was first proposed in 2012, numerous studies have reported that ferroptosis plays a role in a variety of human diseases and is closely associated with many pathological processes (Li S. et al., 2024). A number of precision medicine approaches leveraging ferroptosis have emerged, such as radiotherapy, nanocarrier therapy, and pharmacological intervention. From a biological perspective, normal cells possess intrinsic mechanisms to restrain ferroptosis and sustain homeostasis, thereby preserving regular cellular metabolism.

### 2.1 Initiation of ferroptosis

Ferroptosis initiation is dependent on bioavailable labile iron, which drives cell death via two core biochemical cascades. Firstly, iron ions act as catalysts to initiate the Fenton reaction, catalyzing the reaction of hydrogen peroxide to highly reactive hydroxyl radicals (·OH). Secondly, iron serves as a cofactor of lipoxygenases (LOXs) to participate in lipid peroxidation and is involved in cytochrome P450-mediated peroxidation reactions. Polyunsaturated fatty acids (PUFAs, such as arachidonic acid AA, adrenic acid AdA) are activated and translocated by the reaction of acyl-CoA synthetase long-chain family member 4 (ACSL4) and lysophosphatidylcholine acyltransferase 3 (LPCAT3), which esterify PUFAs to CoA. The resulting PUFAs-PL are incorporated into the sn-2 position of phosphatidylethanolamine (PE) in the biomembranes, rendering them susceptible to catalysis by key enzymes. At the early stage of ferroptosis, mitochondrial membrane and endoplasmic reticulum membrane are predominant sites, rather than plasma membrane. Fe2^+^ is both a cofactor for ALOXs and a catalyst of the Fenton reaction, making it as a critical trigger in the initiation of ferroptosis.

In addition, iron metabolism not only contributes to the initiation phase of ferroptosis, but also participates in diverse pathological process. For example, iron and calcium signaling form an “iron-ferroptosis-calcium regulatory axis” that is involved in the progression of cognitive impairment in neurodegenerative diseases (Gleitze et al., 2021). Iron also interacts synergistically with various metabolic pathways to affect ferroptosis (Guo D. et al., 2025). At present, iron chelation strategies are also evolving from single iron depletion to strategies that target oxidative stress or immunity (Kourti and Kontoghiorghes, 2025). These strategies exert effects beyond detoxification, and remodel iron homeostasis into the hub for multi-dimensional biological regulation.

### 2.2 The core process of ferroptosis

Excessive ROS attacks Polyunsaturated fatty acid phospholipids (PUFAs-PL) in phospholipid membranes and directly triggers lipid peroxidation. PUFAs-PL, as the core substrates of peroxidation, contain a methylene group (-CH_2_-) adjacent to the double bonds with high electron cloud density. Such structure is prone to be oxidized and catalyzed by LOXs to generate lipid radicals (PUFAs-PL·). PUFAs-PL· reacts with molecular oxygen (O_2_) to generate a lipid peroxyl radical (PUFAs-PL-OO ·). Highly reactive in nature, this product can abstract hydrogen atoms (H) from neighboring molecules to generate lipid hydroperoxides (PUFAs-PL-OOH) and new lipid free radicals. This process is accompanied by the oxidation of adjacent molecules, forming a positive feedback loop and initiating a chain reaction. In the non-enzymatic peroxidation, free iron works as the catalyst to drive the generation of ·OH from hydrogen peroxide (H_2_O_2_), and ultimately to generate nonspecific PUFAs-PL-OOH. The excessive accumulation of PUFAs-PL-OOH severely impairs the physiological function of biomembranes and serves as a decisive determinant of cell fate. The expansion of lipid hydroperoxide (L-OOH) ultimately dictates cell survival or death. To date, consensus has not been reached on how different lipid species modulate ferroptosis (Chen et al., 2026). Accordingly, identifying novel lipid-specific targets may represent a promising therapeutic strategy. For example, targeting mitochondria-associated endoplasmic reticulum membranes (MAMs) has been validated to reshape lipid metabolism to drive ferroptosis, considering the critical roles of mitochondria and endoplasmic reticulum in the pre-ferroptosis stage (Zhang Z. et al., 2024).

### 2.3 Defense mechanisms of ferroptosis

Multiple intracellular mechanisms participate in the clearance of toxic free radicals and lipid peroxides. Currently, these mechanisms are broadly classified into GPX4-dependent and GPX4-independent pathways. The glutathione (GSH)-glutathione peroxidase 4 (GPX4) axis serves as the primary regulatory pathway. GPX4 catalyzes GSH-mediated conversion of lipid hydroperoxides (L-OOH) into nontoxic PUFAs-PL-OH. This process blocks the core propagation step of ferroptosis and suppresses lipid chain reactions, thereby markedly slowing ferroptotic progression. At the same time, accumulated ROS also depletes intracellular GSH and further raises lipid peroxide levels.

GPX4-independent regulatory pathways are increasingly recognized. Dihydroorotate dehydrogenase (DHODH), located in the inner mitochondrial membrane, traditionally functions in the synthesis of pyrimidines. Recent evidence reveals that DHODH reduces coenzyme Q (CoQ, CoQ10) to dihydrocoenzyme Q (CoQH2), and can cooperate with GPX4 pathway in mitochondria to inhibit mitochondrial membrane lipid peroxidation. FSP1, which reduces CoQ to CoQH2 with NADH and NADPH serving as electron donors, functions as an oxidoreductase. CoQH2 directly scavenges lipid peroxyl radicals within biological membranes. This pathway becomes dominant upon GPX4 pathway dysfunction. Tetrahydrobiopterin (BH4) is a key intracellular antioxidant, and its synthesis relies on guanosine triphosphate cyclohydrolase 1 (GCH1). BH4 serves as an essential cofactor for aromatic amino acid hydroxylases and nitric oxide synthases. Beyond its cofactor activity, BH4 also exerts direct cytoprotective effects, including antioxidant capacity and mitochondrial function regulation. Notably, the biological functions of this pathway appear inconsistent across tumor types. Existing studies have reported that METTL3 transcriptionally upregulates GCH1 and elevates BH4 level via PBX1 in gastric cancer, thereby facilitating tumor progression and lymph node metastasis (Liu et al., 2022). In hepatocellular carcinoma (HCC), GCH1 expression is frequently downregulated via promoter methylation. Restoration of GCH1 expression enables BH4 to limit superoxide anion accumulation and to block the ASK1/p38 signaling cascade, thus suppressing tumor growth (Zhong et al., 2021). Such tumor-type-dependent functional divergence suggests that the function of signaling pathways is not intrinsic but profoundly shaped by the intrinsic characteristics of the cell and the regulation of the microenvironment.

The integrated mechanism of ferroptosis is mainly divided into three interrelated modules: iron metabolism, lipid peroxidation, and SLC7A11/GPX4 antioxidant defense line. Transferrin carries Fe^3+^ into the cell through TfR1, which is reduced by STEAP and transported by DMT1 to form an intracellular free iron pool LIP. Iron regulatory proteins (IRP1/2) determine TfR1 mRNA activity to regulate intracellular iron abundance. Besides, ZIP8 (SLC39A14) and ZIP14 (SLC39A8) mediate the non-specific uptake of non-transferrin-bound iron (NTBI). This pathway is frequently activated under iron overload and inflammatory conditions (Pasquadibisceglie et al., 2023). Free Fe^2+^ can produce ROS through Fenton reaction and act as lipoxygenase coenzyme to promote lipid peroxidation. FPN1 can expel intracellular iron, inhibit iron accumulation and oxidative stress. Hepcidin can inhibit the activity of FPN1, indirectly promote ferroptosis, and NCOA4 mediated ferritinophagy degrades ferritin, further increasing free iron level. ACSL4 and LPCAT3 activate PUFAs and integrate them into the cell membrane, providing a substrate for lipid peroxidation. ROS and lipoxygenase continue to catalyze the generation of toxic lipid hydroperoxides and continue to promote the ferroptosis process. System Xc^−^, composed of SLC7A11 and SLC3A2, transports cystine into the cell to provide raw materials for GSH synthesis. Ferroptosis inducers, such as Erastin and RSL3, act on the System Xc- to trigger ferroptosis by depleting intracellular GSH. GPX4 depends on GSH to remove lipid peroxides, and the whole pathway inhibits ferroptosis. Once the function of SLC7A11, GSH or GPX4 is impaired, the cellular antioxidant barrier breaks down, and the iron and ROS-induced lipid damage will not be limited. The oxidative stress caused by iron accumulation and the antioxidant capacity of the GPX4 pathway form a dynamic balance. The integrated mechanism of ferroptosis is shown in Figure 1.

**FIGURE 1:**
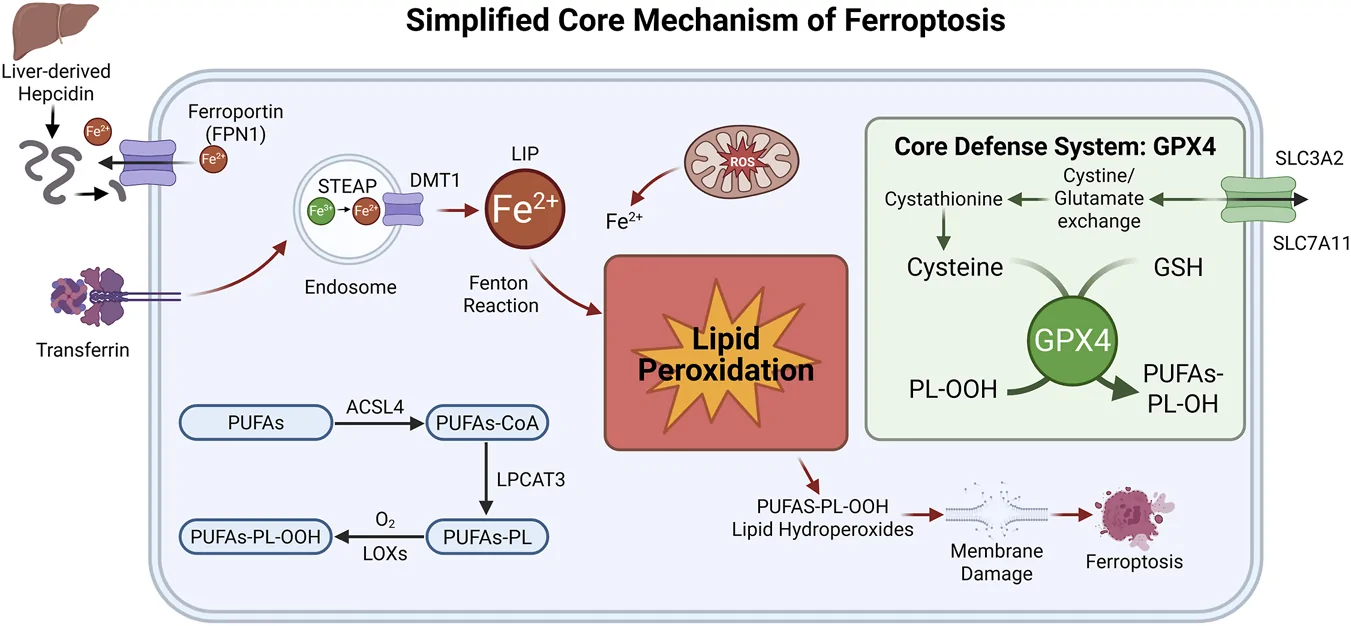
Fe^3+^ bind to transferrin and enter the cell through TfR1 to form endosomes. STEAP converts Fe^3+^ to Fe^2+^, which then enters the cytoplasm via DMT1 to form LIP. Some Fe^2+^ from mitochondria also enters the cytoplasm. Fe^2+^ in the LIP participates in the Fenton reaction to promote lipid peroxidation. PUFAs are gradually converted into lipid peroxides through ACSL4, LPCAT3, and LOXs. Lipid peroxides cause damage to the plasma membrane and promote ferroptosis. The core defense system within the cell is the GPX4-GSH axis. With the assistance of System Xc^−^, cystine, as a raw material for GSH, enters the cell and converts L-OOH into PL-OH. The left part of the figure represents the iron metabolism module, the middle red part represents the cascade of lipid peroxidation, and the right green part represents the main antioxidant defense system. Black solid arrows represent material transport/enzyme catalyzed forward reactions. Bold red arrows represent the pro-ferroptosis toxicity cascade. Solid green arrows represent the anti-ferroptosis antioxidant defense cascade. The red explosion icon represents the central reaction hub of lipid peroxidation. The red fragmented cell membrane represents the ferroptosis terminal membrane rupture phenotype. Extracellular side represent cystine/glutamate gradient.

In addition, emerging evidence reveals that multiple metabolic factors can modulate ferroptosis. Selenium enrichment increases cellular intrinsic resistance to ferroptosis. As a negative regulator of ferroptosis, peroxiredoxin 6 (PRDX6) suppresses ferroptosis by enhancing selenium bioavailability and promoting GPX4 synthesis (Ito et al., 2024).Selenium also regulates ferroptosis through a variety of pathways (Arisawa et al., 2025; Xia et al., 2026). MJ33, bilirubin and vitamins A/E uniformly suppress lipid peroxidation and ferroptosis, yet their clinical translation is hindered by non-specific systemic metabolic toxicity and insufficient tissue targeting precision (Jakaria et al., 2023; Torres-Velarde et al., 2024; Noguchi et al., 2025; Wang J. et al., 2025).

### 2.4 Mechanisms regulating ferroptosis

Iron homeostasis profoundly affects ferroptosis sensitivity (Guo et al., 2024; Lee et al., 2025). Mitochondria serve as the primary site for heme and iron-sulfur cluster synthesis and require large amount of iron. Unlike cytoplasmic iron metabolism, mitochondria possess an independent iron regulatory system. Mitochondrial iron uptake relies on transporters such as Mitoferrin-1/2 (MFRN1 and MFRN2), which are essential for sustaining mitochondrial iron homeostasis (Ali et al., 2022). Mitochondrial ferritin (MtFer) sequesters excess Fe^3+^. Iron deficiency impairs mitochondrial function by disrupting the electron transport chain (ETC.), particularly complexes I, II and III that contain iron-sulfur clusters. Current evidence indicates that SELENOK deficiency reduces intracellular iron content, induces aberrant TfR1 palmitoylation, and ultimately disrupts mitochondrial function in neurons (Jia et al., 2025).

Mitochondria serve as the core hub for metabolic regulation, oxidative stress control, morphological maintenance and cell fate determination, critically shaping intracellular redox status. Their primary physiological function relies on the tricarboxylic acid (TCA) cycle ETC., which are the major sources of endogenous ROS production. During ferroptosis, dysfunction of mitochondrial respiratory chain complexes I and III triggers electron leakage and excessive superoxide anion (O2·-) production, further driving lipid peroxidation. Massive radical accumulation in the late phase of ferroptosis marks an irreversible stage of cell death (Bow et al., 2023). On the other hand, mitochondria represent a major site of lipid peroxidation. Peroxidation of PUFAs in the mitochondrial membrane greatly accelerates ferroptosis. Accumulating evidence has confirmed that ER-mitochondrial contact sites (MAMs) are enriched in polyunsaturated phospholipids and serve as the primary initiation site of phospholipid peroxidation. Expansion of these contact regions enhances mitochondrial ROS generation and mitochondrial fission, thereby intensifying ferroptosis. Moreover, MAMs regulate ferroptosis through calcium trafficking, which remodels cellular lipid composition and alters the progression of lipid peroxidation (Zhang Z. et al., 2024). Mitochondria function as a central hub for ferroptosis regulation and harbor multiple intrinsic defense mechanisms. Beyond the DHODH-CoQ axis, mitochondrial superoxide dismutase 2 (SOD2) converts matrix superoxide anions into hydrogen peroxide. This product is subsequently broken down by glutathione peroxidase1 (GPX1) and catalase (CAT) to sustain stable ROS balance. Mitochondrial complex I (MC I) also suppresses ferroptosis by maintaining physiological NADH and CoQ levels. This protective effect can be abolished by the complex I inhibitor IACS-010759. Notably, this regulatory pathway operates independently of both the GPX4 and FSP1 axes, offering a novel therapeutic strategy targeting MC I for ferroptosis modulation (Deng et al., 2025). In addition, distinct changes in mitochondrial morphology are also observed during ferroptosis, manifested as swelling or reduced volume. The number of cristae structures decreased or disappeared, and even fragmentation occurred (Su et al., 2025). Structural damage of this kind is considered to weaken cellular defenses against ferroptosis (Rai et al., 2024). This is because mitochondrial cristae serve as hubs for energy metabolism, iron-sulfur cluster synthesis and lipid metabolism regulation. Wang’s research further highlights the vital role of intact mitochondrial ultrastructure (Wang Z. et al., 2024). Changes in mitochondrial morphology may not only reflect the occurrence of ferroptosis, but also actively participate in regulating this process. Impaired mitochondrial fusion (resulting from downregulated OPA1 expression) promotes ferroptosis, while treatment with TRPA1 agonists restores mitochondrial morphology and alleviates ferroptosis (Weng et al., 2024). Targeted intervention in mitochondrial morphology to precisely induce ferroptosis may become a promising research direction in the future. Furthermore, during ferroptosis in A549 cells, mitochondrial viscosity increases significantly from 43.58 cP to 152.05 cP, indicating dramatic changes in the internal physical state of mitochondria and profound interference with ferroptosis progression (Li et al., 2023). Metabolic disorders of high-energy compounds such as ATP and ADP in the mitochondrial membrane may activate AMPK, eventually upregulating FSP1 and inhibiting ferroptosis (Ma S. et al., 2025).

The structure and composition of biomembranes themselves profoundly influence the occurrence and progression of ferroptosis. Studies have demonstrated that compared with non-conjugated PUFAs, conjugated PUFAs exhibit a more potent capacity to promote ferroptosis, and this mechanism is associated with the formation of reactive electrophilic aldehydes (Do et al., 2023). Furthermore, the integration of these fatty acids into distinct lipid molecules exerts different effects on ferroptosis susceptibility. Several research groups have reported that monounsaturated fatty acids can inhibit ferroptosis by reducing phospholipid peroxidation, whereas certain long-chain saturated fatty acids tend to promote ferroptosis (Cui and Ye, 2026). Collectively, the molecular weight, saturation level, and chemical structure of fatty acids act as critical determinants governing ferroptosis. Membrane microdomains are dynamic regions composed of lipids and proteins within biomembranes, whose functions vary depending on the cellular environment. As typical membrane microdomains, lipid rafts are enriched in cholesterol and sphingolipids. They can spatially restrict the diffusion of lipid peroxides, thereby exerting an inhibitory effect on ferroptosis. In tumor models, disruption of lipid raft structure by the SOAT1 inhibitor Avasimibe, combined with auranofin, promotes massive accumulation of lipid peroxides and suppresses tumor growth (Han et al., 2026). In addition, enzymes and transporters in biomembranes also constitute a complex regulatory system that affects the cellular functions of cells. Plasma membrane rupture is a central event in cell death, yet the underlying mechanisms remain incompletely understood. Current evidence suggests that oxidized lipids may increase membrane tension and activate mechanosensitive cation channels (such as Piezo1 and TRP family members), thus exacerbating ion dyshomeostasis (Hirata et al., 2023). Alternatively, oxidized lipids may alter the physical properties of the membrane, leading to reduced fluidity and a decrease in the abundance of ordered membrane domains (e.g., lipid rafts).

Ferroptosis denotes an iron-dependent subtype of regulated cell death, where lipid peroxidation constitutes its dominant cytotoxic cascade. Cells counteract ferroptosis through multiple intrinsic defense mechanisms to maintain homeostasis, and ferroptosis is dynamically regulated by a variety of molecules and metabolic pathways. As a promising therapeutic target in oncology, ferroptosis has attracted extensive attention in oncology. RT is a primary treatment modality for lung cancer. Ionizing radiation (IR) eliminates tumor cells by inducing ROS outbursts, DNA lesions and mitochondrial dysfunction, biological events that overlap substantially with the core mechanisms of ferroptosis. In fact, ferroptosis itself is one of the important mechanisms by which radiotherapy exerts anti-tumor effects, making the combination of ferroptosis and radiotherapy expected to become an important part of the comprehensive treatment of lung cancer. Lung cancer is a highly heterogeneous tumor, with distinct subtypes such as NSCLC and small cell lung cancer (SCLC) showing significant differences in radiotherapy sensitivity. However, it remains unclear whether specific gene mutations (such as EGFR mutation and LKB1 loss) modulate radiotherapy responses through ferroptosis-related mechanisms. Therefore, it is essential to explore how radiotherapy modulates the ferroptosis pathway in lung cancer cells. In particular, different lung cancer subtypes and specific biological contexts significantly affect cellular responses to IR induced ferroptosis, including genetic mutations, non-coding RNAs (ncRNAs), and protein post-translational modifications (PTMs). Further investigation in these directions can expand mechanistic understanding and yield actionable clues for enhancing radiosensitivity and overcoming radioresistance in lung cancer.

## 3 Mechanism of radiotherapy-induced ferroptosis

Radiotherapy is one of the standard therapeutic modalities for advanced malignant tumors. IR is widely recognized to directly induce DNA double-strand breaks, triggering cell death in forms such as pyroptosis or apoptosis (Ning et al., 2024). Radiation induced vascular endothelial damage leads to intratumoral microthrombosis, ischemia and hypoxia, while simultaneously triggering tumour cell senescence. (Jiang et al., 2026). Moreover, IR can decompose water molecules to generate massive hydroxyl radicals, induce lipid peroxidation in tumor cells, and downregulate ferroptosis suppressor genes—thus making ferroptosis another lethal mechanism of RT (Zhang et al., 2021). Strictly speaking, there are relatively few studies focusing on radiotherapy-induced ferroptosis directly in lung cancer cells. However, a clear mechanistic pathway has been established in recent years. Moreover, numerous studies have demonstrated that various drugs and bioactive molecules modulating ferroptosis pathways can enhance tumor radiosensitivity (Table 1).

**TABLE 1:** Summary of molecules that enhance radiosensitivity via ferroptosis.

Molecule	Cell lines	Class and mechanism	References
DFO	A549 H1299	Iron Chelator DNA break and depletion of LIP	(Bayanbold et al., 2023)
Doxycycline	A549 H1299	Tetracycline antibiotics DNA break and depletion of LIP and delaying in S-phase transit	(Bayanbold et al., 2023)
DNSP (NaYF4Yb/Er@NaGdF4)	A549 H1299	Downshifting nanoparticles Degradation of SPARC and inhibition of GPX4 and SLC7A11 to enhance ferroptosis and radiosensitivity	(Aishajiang et al., 2026)
Hemin	A549 H460 H1299 MRC5 HFL1 Calu-1	Lung cancer cells: iron overload and induction of ROS and GPX4 degradation Normol cells: upregulation of FTH1 and stable iron metabolism and ROS level	(Almahi et al., 2022)
BIBR1532	A549 H460 LLC-OVA	telomerase inhibitor cGAS-STING pathway activation	(Bao et al., 2024)
Soybean lecithin–gallic acid complex	A549 H1299 BEAS-2B	ROS generation and mitochondrial damage Inhibition of the Nrf2/SLC7A11/GPX4 axis	(Chen et al., 2025a)
DHA-N@M	LLC MLE-12	PUFA-Based nanoreactor NO reacts with ROS to generate ONOO- DHA induced disorders of lipid metabolism	(Chen et al., 2025b)
PHKG2	A549 NCI-H358 NCI-H2170 SK-MES-1	Protein PHKG2 overexpression leads to elevated intracellular iron levels by promoting ferritinophagy and mitochondrial stress-dependent ferroptosis	(Han et al., 2024)
Glutaminase	A549 NCI-H123 HCC827 NCI-H226 LLC	Protein GLS silence inhibits DSB repair and promotes ferroptosis	(Jiang et al., 2025a)
MAFF	A549 and its cisplatin-resistant subline BEAS-2B HPAEpiC	MAF bZIP transcription factor F MAFF regulates SLC7A11 to promote ferroptosis and radiosensitivity Ionizing radiation can upregulate MAFF through CAMP/PKA/CREB1	(Liang et al., 2024a)
Nortriptyline hydrochloride	A549 NCI-H1299 HEK293T	RBMS1 inhibitor Nortriptyline hydrochloride inhibited RBMS1 expression and SLC7A11 to promote ferroptosis and radiosensitivity	(Zhang et al., 2021)
FBLN5	A549 LLC NIH3T3 MRC5 CAFs/NFs	Protein Knockdown of FBLN5 promotes ROS production and MDA to improve ferroptosis and radiosensitivity after radiotherapy. FBLN5 enhances radiation resistance through Src-STAT3-ACSL4 signaling axis and exosomes	(Zhang et al., 2025c)
Cabozantinib	A549 H1299	Tyrosine Kinase Inhibitor (TKIs) Cabozantinib increases the radiosensitivity of NSCLC cells by inhibiting ferroptosis induced by STAT3/MCL1/BECN1/SLC7A11 axis	(Wang et al., 2025a)
Clobetasol propionate	A549	Nrf-2 inhibitor Clobetasol propionate, combined with radiotherapy, can enhance mitochondrial ROS and iron abundance as well as lipid peroxidation to improve radiosensitivity	(Rai et al., 2024)
MGST1	NCI = 1299-IR HCC827-IR	microsomal glutathione s-transferase 1 MGST1 enhances the radiosensitivity of NCSLC cells by promoting ALOX15 -mediated ferroptosis by regulating the HO-1/DNMT1 pathway	(Ma et al., 2025b)
PS-MPs	A549	Polystyrene microplastics After ionizing radiation, PS-MPs significantly attenuated radiation-induced ferroptosis and activated the NF-κB pathway	(Zhou et al., 2026)

### 3.1 Evidence of ferroptosis induced by radiotherapy

Radiotherapy is thought to act primarily by triggering apoptosis. However, clinical observations show that the efficacy of radiotherapy is not fully consistent with the apoptotic sensitivity of tumor cells, implying the involvement of alternative cell death mechanisms or the presence of apoptosis resistance. Previous studies have observed typical ferroptotic characteristics in lung cancer cell lines exposed to IR, including mitochondrial damage and altered morphology, excessive ROS accumulation, lipid peroxide buildup, and upregulated expression of the ferroptosis marker gene PTGS2. This directly confirms that ferroptosis contributes to radiation-induced tumor cell death. It was previously believed that the apoptosis dominates over ferroptosis when the tumor cells are destroyed by IR. However, pharmacological inhibition experiments have shown that ferroptosis inhibitors provide a stronger protective effect than apoptosis inhibitors (Ren et al., 2020). Combined with the differences in the core regulatory mechanisms between ferroptosis and apoptosis, the essence of this phenomenon may be that the initiation efficiency and regulatory dependence of the 2 cell death pathways are different. Ferroptosis depends on the efficient chain reaction of lipid peroxides, and this chain reaction relies on adjacent molecules to supply hydrogen atoms. Such physically neighboring interactions greatly accelerate reaction initiation, enabling ferroptosis to be preferentially triggered and to dominate ionizing radiation-induced cell death under specific conditions. In contrast, apoptosis is executed via cascade signaling, an ATP-consuming process that proceeds at a relatively slower kinetic rate. This is not a unique phenomenon. Another study has found that ferroptosis is more important than apoptosis under certain conditions, even during radiotherapy (Xu et al., 2025).In addition, based on the data of 1727 NSCLC patients in TCGA and GEO databases, it is reported that the ferroptosis score is significantly correlated with the positive response of patients to radiotherapy (P < 0.001), suggesting that radiotherapy may play a role by inducing ferroptosis (Zhang N. et al., 2022). In another study, radioresistant NSCLC cell lines were constructed. Among various cell death inducers, only ferroptosis inducer could eliminate the biological differences between drug-resistant cells and parental cells. Compared with GPX4, FSP1 knockout can significantly reverse the radiation resistance to the level of primary cells (Lin et al., 2024). This phenomenon, together with the abnormal upregulation of CoQ synthesis in metabolism, shows a GSH-independent radioresistance pathway mediated by the FSP1-CoQ axis, which provides direct evidence for the elimination of lung cancer cells by ferroptosis via radiotherapy. At the same time, many studies combined with ferroptosis inducers can synergistically improve ferroptosis level, which also indirectly supports above viewpoint (Ye et al., 2020; Chen Y. et al., 2025). Based on the *in vitro* experiment, data analysis and mechanism exploration, ferroptosis acts as a prominent complementary cell death pathway alongside apoptosis, and participates in shaping cellular radiosensitivity and therapeutic resistance phenotypes.

### 3.2 Radiotherapy-regulated ferroptosis network

Radiotherapy-induced ferroptosis in lung cancer cells also mainly involves iron metabolism, progressive lipid peroxidation and dysfunction of antioxidant mechanisms (Vilaplana-Lopera et al., 2025).Based on the available literature, this section outlines the regulatory roles of emerging molecular pathways, such as gene mutations, RNA and post-translational modification (PTMs). Radiotherapy-induced lipid peroxidation can induce ferroptosis (Zhang L. et al., 2024). Although some studies have not directly explored the association between ferroptosis and radiosensitivity, elevated antioxidant capacity and the ability to clear lipid peroxides can also confer radioresistance via inhibiting ferroptotic signaling (Zhao M. et al., 2023; Rai et al., 2024; Wang D. et al., 2024).

Radiotherapy disrupts intracellular iron homeostasis and triggers iron-dependent oxidative damage. Clinical studies have found significant changes in iron metabolism markers in patients receiving radiotherapy (Han et al., 2024; Hua et al., 2025; Jin et al., 2025). Lung cancer cells display enhanced iron dependency compared with normal cells (Sukiennicki et al., 2019; Bayanbold et al., 2023), rendering them more vulnerable to iron metabolism disorders. Doxycycline and DFO improve the chemoradiation sensitivity of lung cancer cells by reducing LIP levels (Bayanbold et al., 2023). Besides, radiotherapy disturbs intracellular iron balance by regulating key iron metabolism-related genes. For instance, radiotherapy relieves Nrf2 mediated transcriptional repression of PHKG2, thereby increasing PHKG2 expression. This process promotes ferritinophagy and mitochondrial dysfunction, and ultimately enhances ferroptosis and radiosensitivity in NSCLC (Han et al., 2024) (Figure 2). Targeting Nrf2 can block PHKG2 expression upstream. Although ionizing radiation generally elevates intracellular labile iron levels, the iron regulatory network features multilayered complexity. To date, no consistent consensus has been reached regarding whether TFR1 and other iron membrane transporters undergo expression fluctuations post-irradiation, which requires further mechanistic validation. Reducing sensitivity to autophagy-dependent ferroptosis may be another way to regulate radioresistance. It has been reported that the HNRNPA2B1/HDGF/PTN signaling pathway participates in the regulation of autophagy and ferroptosis in radioresistant lung cancer cells (Han et al., 2025). HNRNPA2B1 also modulates ferroptosis via distinct mechanisms. For example, it specifically binds to m6A modification sites on LDHA mRNA and drives lactate accumulation, which in turn confers antioxidant protection. *In vitro* co-culture studies have shown that elevated HNRNPA2B1 expression attenuates CD8^+^ T cell-mediated tumor clearance and directly suppresses ferroptosis (Zhang Y. et al., 2025).

**FIGURE 2:**
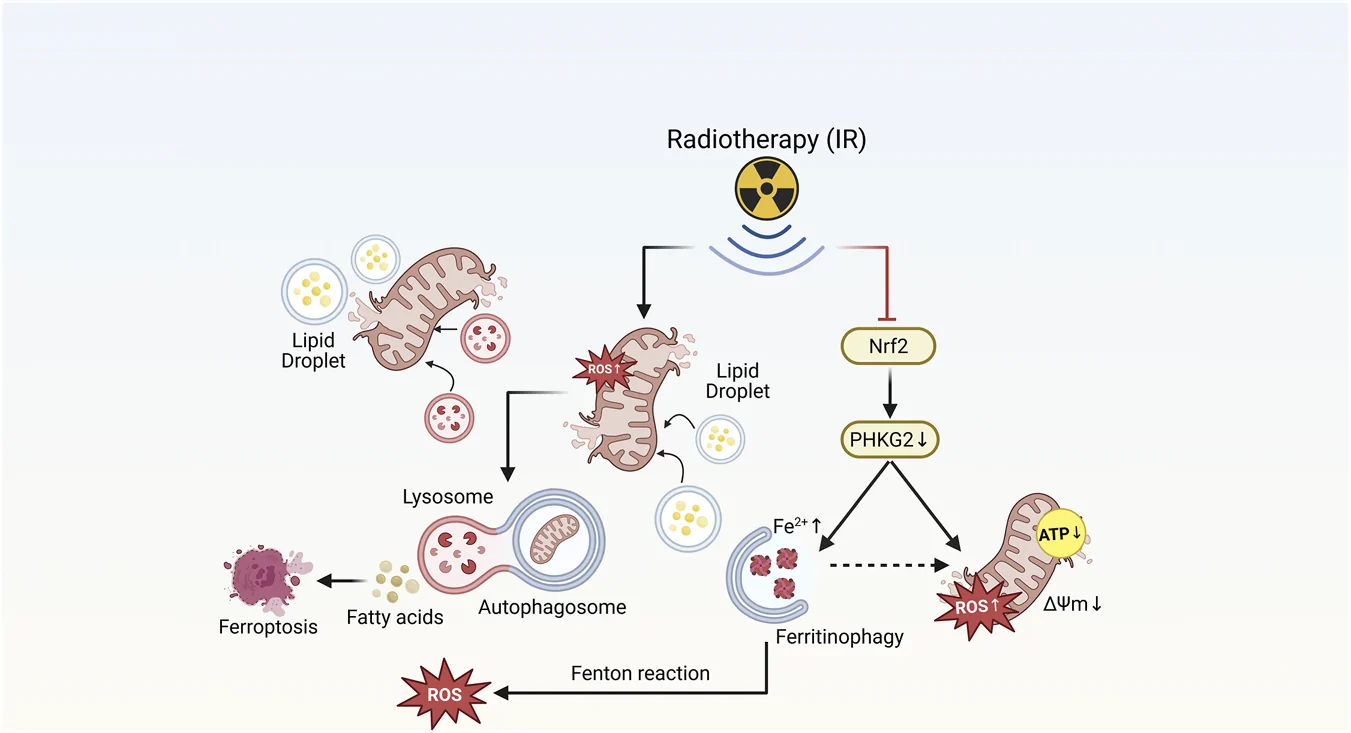
Mitochondrial damage caused by radiotherapy is divided into two processes. Radiotherapy causes massive synthesis of lipid droplets and surrounds damaged mitochondria. Due to mitophagy, lysosomes phagocytose mitochondria and lipid droplets, releasing free fatty acids and promoting ferroptosis. Radiotherapy inhibits Nrf2 and increases PHKG2 expression, leading to ferritin autophagy, mitochondrial dysfunction and decreased membrane potential. An increase in free iron may also exacerbate mitochondrial dysfunction. Yellow radiation symbol represents ionizing radiotherapy stimulus. Yellow circles represent fatty acids. Black solid arrows represent physiological substance trafficking and signal transmission. Red arrows represent oxidative stress/ferroptosis-promoting signaling. Black dashed arrows represent speculative interactions. Red star/red burst icons represent excessive ROS. Downwards black triangle symbol represents decreased mitochondrial membrane potential (ΔΨm). Pink irregular shapes represent ferroptosis phenotype. The red flathead line represents inhibition. Gray circles represent lipid droplets.

ROS produced by ionizing radiation damages mitochondria and drive massive lipid droplet accumulation around the damaged mitochondria. Ultimately, this can trigger mitophagy. Lysosomes phagocytose mitochondria and lipid droplets and finally excrete fatty acids to facilitate ferroptotic progression (Yang et al., 2023) (Figure 2). In addition, radiotherapy remodels lipid metabolism in lung cancer cells and increases the production of lipid peroxides such as malondialdehyde, thus promoting lipid droplet accumulation. Photodynamic therapy (PDT) toward specific lipid droplets can effectively trigger ferroptosis in synergy with radiotherapy, thereby overcoming acquired tumor radioresistance (Tirinato et al., 2021; Xu S. et al., 2026).

Numerous studies have demonstrated that radiotherapy represses the activity of key antioxidant enzymes through multiple pathways. For instance, IR inhibits GPX4 activity and further downregulates SLC7A11. Furthermore, research has shown that low-dose radiotherapy combined with PD-1 inhibitors exacerbate oxidative stress by blocking the Nrf2/HO-1/GPX4 axis, thus triggering ferroptosis in lung cancer cells (Luo et al., 2025) (Figure 3). Moreover, radiotherapy upregulates dihydroorotate dehydrogenase (DHODH) via a CREB1-dependent mechanism. DHODH restrains ferroptosis and boosts DNA damage repair capacity by producing ubiquinol, thereby promoting the emergence of radioresistant phenotypes (Mao et al., 2026) (Figure 3).

**FIGURE 3:**
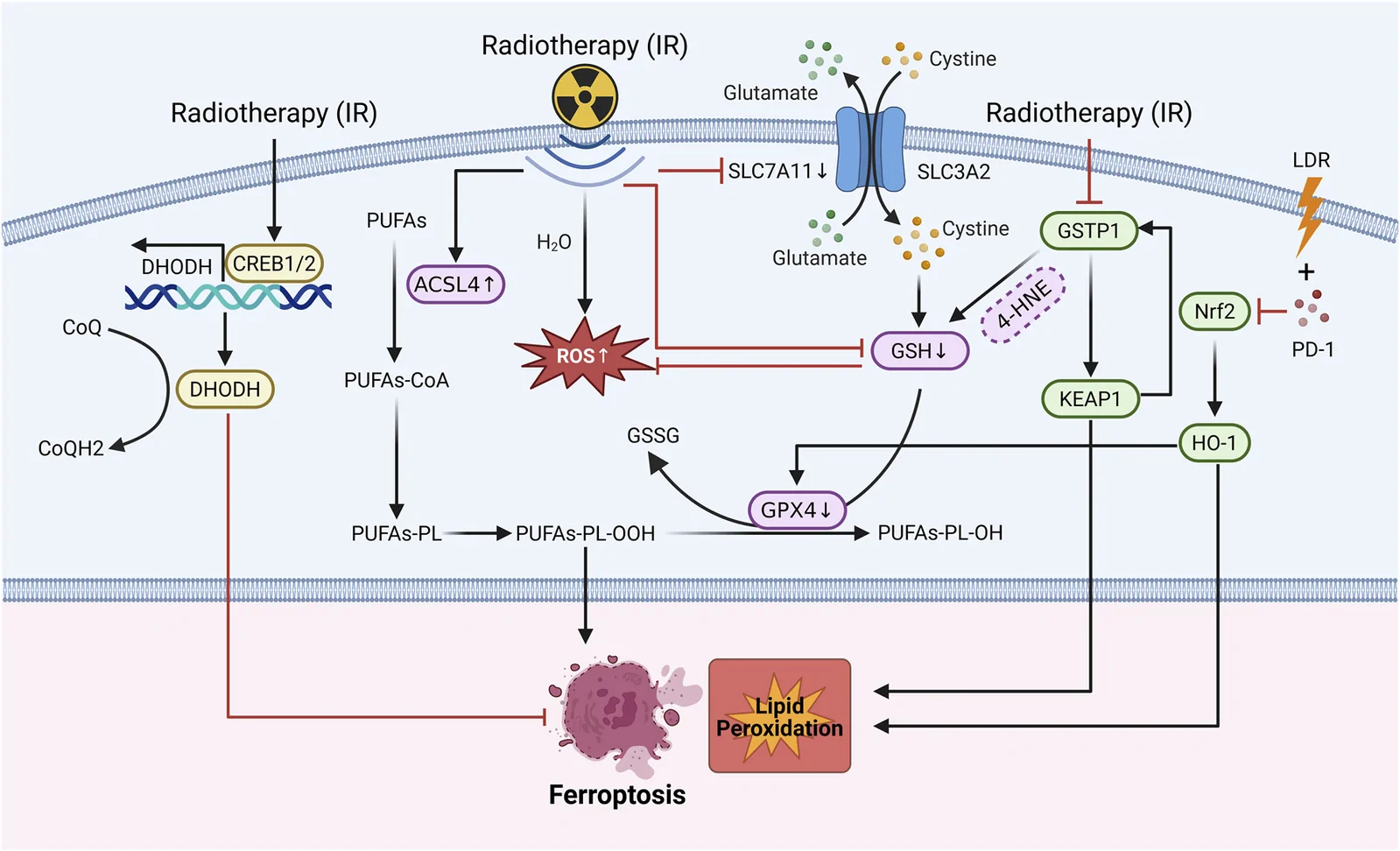
RT can decompress water molecules to generate a large amount of ROS. Meanwhile, RT can also promote ACSL4 and downregulate SLC7A11. ACSL4 is a key enzyme in the lipid peroxidation process. Inhibition of SLC7A11 results in a decrease in System Xc^-^transporting cystine, resulting in a decrease in GSH. GSTP1 is used to remove lipid peroxides, and it can react GSH with 4-HNE to remove toxic peroxides. GSTP1 can be inhibited by radiotherapy and also has the function of activating KEAP1. KEAP1 in turn activates GSTP1 via the Keap1-Nrf2 axis. This process forms a positive feedback pathway. LDR in combination with PD-1 inhibits Nrf2 and downregulates GPX4 via HO-1, leading to lipid peroxidation. Radiotherapy activates the transcription factor CREB1/2 and enhances the expression of DHODH, leading to increased CoQH2 production. The ferroptosis pathway was inhibited. The light blue band represents the bilayer plasma membrane. Cystine/glutamate gradient is represented on the outer side of the cell. Yellow radiation mark represents IR stimulation. Black arrows represent physiological substance transitions and signaling as well as forward responses. The red flathead line represents inhibition. Yellow and green far points represent cystine and glutamate.

Glutathione S-transferase P1 (GSTP1) is widely distributed and highly expressed in lung cancer tissues, which functions as an endogenous anti-ferroptotic mediator. It directly eliminates lipid peroxides through the GPX4-independent pathway and catalyzes the reaction of GSH with the product of lipid peroxides (4-HNE) (Zhang W. et al., 2023). Notably, GSTP1 expression is downregulated in cells after low-dose radiotherapy (Cui et al., 2024), which may enhance radiosensitization by promoting ferroptosis (Tan et al., 2022) (Figure 3). In addition, based on the regulatory relationship between KEAP1, Nrf2 and GSTP1, it has been speculated that GSTP1-KEAP1-Nrf2 positive feedback loop may form to protect lung cancer cells from oxidative damage under ionizing radiation or oxidative stress. To date, no direct preclinical or clinical evidence has validated GSTP1 as a molecular linker bridging radiosensitivity and ferroptosis in lung malignancies, and further mechanistic investigations are required to verify this regulatory axis.

#### 3.2.1 Regulatory mechanisms of gene mutations

Genetic mutations represent a primary driver of lung cancer, with EGFR and KRAS alterations being the most prevalent. Concurrent multiple mutations are also frequently observed, such as combined LKB1 and KEAP1 deficiency alongside KRAS mutation (Pons-Tostivint et al., 2021). With the widespread application of advanced sequencing technologies, more rare molecular subtypes are expected to be identified in the future. Accumulating Evidence has indicated that genetic mutations profoundly affect tumor radiosensitivity and ferroptosis susceptibility. For instance, EGFR-mutant lung cancer cells are highly vulnerable to ferroptosis inducers, while other common subtypes, including KEAP1 or LKB1 loss, also display distinct regulatory patterns of ferroptosis (Koppula et al., 2022; Zhang Y. P. et al., 2026). Large-scale functional screening has revealed that both common and rare gene mutations markedly alter cellular survival following radiation exposure, and can directly drive radioresistance (Gopal et al., 2021; Tanaka et al., 2023b). While targeted therapies have achieved favorable clinical outcomes in major molecular subtypes, the exact mechanism, connecting distinct gene variants to therapeutic responses have not been fully elucidated. This review summarizes frequent gene mutations in lung cancer and discusses how these variants regulate ferroptosis during radiotherapy, aiming to provide mechanistic evidence for precision therapy and rational combination treatment strategies.

Radiotherapy can activate wild-type p53 (WT-p53). By repressing SLC7A11 expression, WT-p53 limits GSH production, thereby augmenting ferroptosis and enhancing radiosensitivity (Lei et al., 2021). In contrast, lung tumors carrying mutant p53 exhibit blunted therapeutic responses to irradiation. The radiosensitivity can be rescued via combinatorial administration of ferroptosis inducers. These findings indicate that the ferroptosis pathway is critical for p53-mediated radiosensitivity. A 2021 study demonstrated that, in p53-null H1299 cells, restoration of p53 after IR markedly elevated levels of lipid peroxides and PTGS2 expression. Meanwhile, cell viability was significantly increased following treatment with ferrostatin-1. Notably, p53 restoration failed to fully reverse radioresistance even when ferroptosis was blocked, suggesting that p53 can improve radiosensitivity through both ferroptosis-dependent and ferroptosis-independent mechanisms. In the organoid model, the team reached the same conclusion (Lei et al., 2021). (Figure 4) Although p53 has no direct regulatory effect on GPX4 enzyme, its activity is barely increased in the face of increased cellular oxidation level. Whether this situation is cell-specific warrants further investigation. It has been reported that the use of drugs to activate the p53 pathway can increase chemotherapy sensitivity, and this study suggests that restoring the function of the p53 pathway in p53-deficiency lung cancer cells may serve as a viable intervention for radiosensitization (Choi et al., 2024).

**FIGURE 4:**
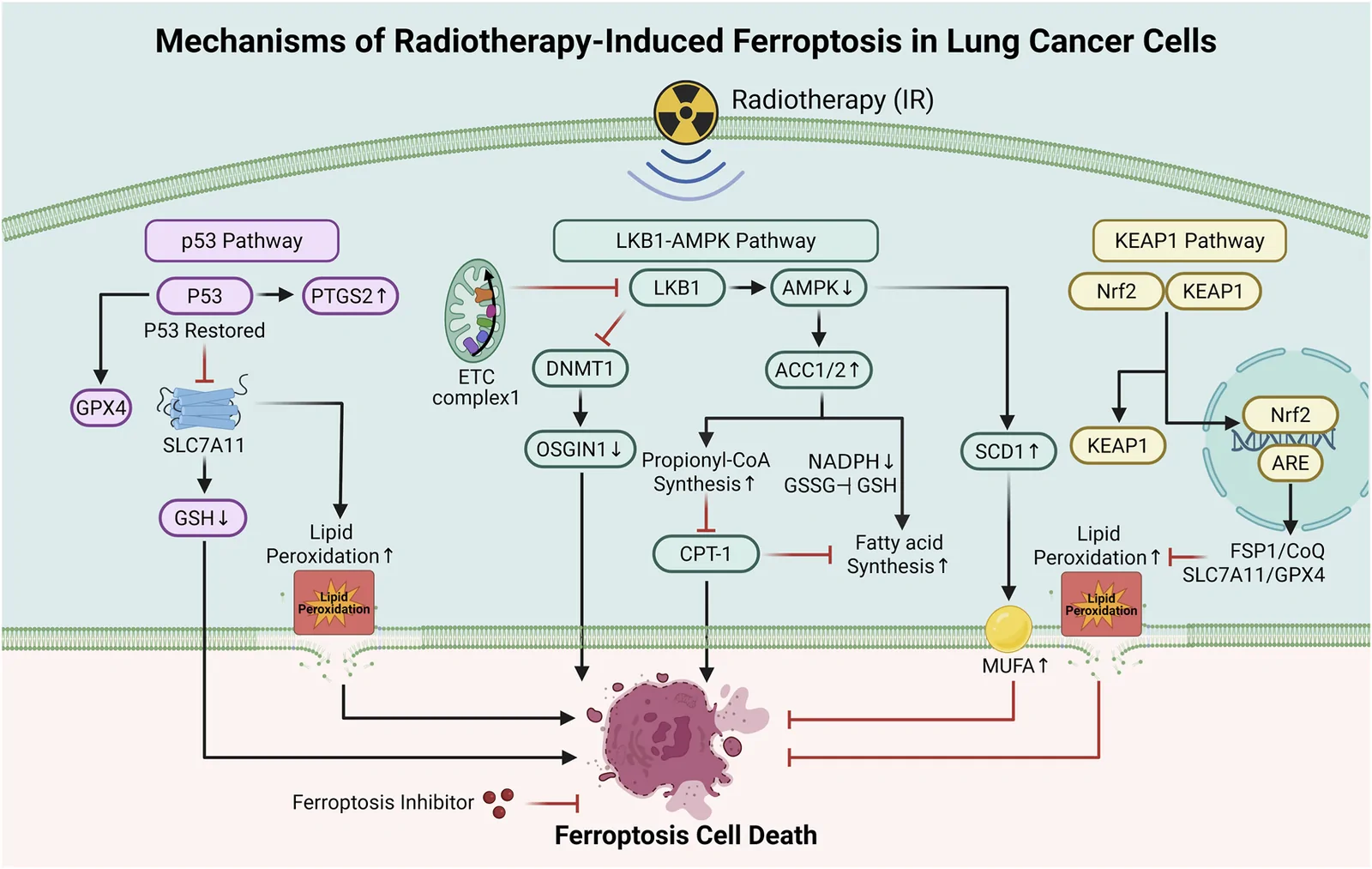
In p53-null lung cancer cells, an increase in PTGS2 and a decrease in SLC7A11 were observed after ionizing radiation and subsequent restoration of P53, leading to ferroptosis due to insufficient GSH synthesis. GPX4 expression was not altered in this experiment. LKB1 loss results in decreased AMPK activity, resulting in increased ACC1/2 and SCD1 activity. At the same time, it causes the disorder of, ETC complexI and the inhibition of DNMT1, which eventually leads to ferroptosis of lung cancer cells. ACC activation promotes cellular fatty acid synthesis, depletes NADPH, fails to convert GSSG to GSH, and finally enhances ferroptosis sensitivity. In addition, activation of ACC1/2 also promoted malonyl-CoA synthesis and inhibited CPT-1 to reduce fatty acid decomposition. LKB1 loss is accompanied by KEAP1 mutations. KEAP1 mutation activates and translocates Nrf2 to the nucleus, where it binds to ARE and upregulates (FSP1/CoQ, SLC7A11 and GPX4) antioxidant genes to inhibit lipid peroxidation and ferroptosis. Solid black arrows indicate physiological material transport and signal transmission. The red flatheaded line represents the inhibition action. Broken cells at the bottom represents ferroptosis cell phenotype. The figure is divided into 3 gene pathways by color from left to right. The purple color represents the P53 pathway. Green represents the LKB1-AMPK pathway. Yellow represents the KEAP1-Nrf2 pathway. Yellow circles represent MUFAs. The small red circles represent ferroptosis inhibitors.

Liver Kinase B1 (LKB1, encoded by STK11 gene) is a serine/threonine kinase that is frequently lost in NSCLC. Cumulative clinical cohort data demonstrate that compared with high expression of LKB1, LKB1 deficiency has a prominent effect on the radiosensitivity of NSCLC (Sitthideatphaiboon et al., 2024). Mechanistically, concurrent loss of LKB1 together with KEAP1 inactivation triggers persistent Nrf2 hyperactivation, which engages a compensatory antioxidant program. This transcriptional cascade attenuates radiotherapeutic efficacy and blunts ferroptotic responses via elevated biosynthesis of monounsaturated fatty acids (MUFAs).

LKB1 acts as the primary upstream kinase responsible for AMPK activation (Schmitt et al., 2022). Accordingly, LKB1-deficient cells exhibit markedly diminished basal AMPK activity yet full AMPK inactivation is rarely observed (Shackelford et al., 2013; Jin et al., 2018). Given the tight functional linkage between LKB1 and AMPK, the two molecules are frequently analyzed as a unified signaling axis. The LKB1-AMPK axis plays a critical role in lipid metabolism, antioxidant effect and regulation of radiosensitivity. Decreased AMPK activity relieves the inhibitory effect of acetyl-CoA carboxylase (ACC1/ACC2), resulting in increased production of malonyl-CoA. In contrast, malonyl-CoA is a potent inhibitor of carnitine palmitoyltransferase 1 (CPT-1), the rate-limiting enzyme in fatty acid β-oxidation. This metabolic shift—characterized by enhanced fatty acid anabolism alongside impaired fatty acid catabolism—triggers massive intracellular fatty acid buildup and indirectly elevates lipid peroxidation to facilitate ferroptosis (Au-Yeung et al., 2023). However, reduced AMPK activity increases stearoyl-CoA desaturase 1 (SCD1) expression. This promotes the synthesis of monounsaturated fatty acids (MUFA) and stabilizes cell membranes, ultimately conferring ferroptosis tolerance (Sen et al., 2023). Similarly, in esophageal squamous cell carcinoma (ESCC), SCD1 mediates radioresistance by inhibiting radiation-induced lipid peroxidation by reducing the synthesis of oleic acid and palmitoleic acid (Luo et al., 2022). Therefore, the LKB1-AMPK axis plays a vital role in regulating fatty acid metabolism and lipid peroxidation. It also mediates the suppression of radiotherapy-induced ferroptosis. In HT-1080 cells, dysfunction of the mitochondrial, ETC complex I can alter radiosensitivity via LKB1-AMPK axis inactivation (Mao et al., 2024) (Figure 4).

To elaborate on how IR modulates ferroptosis, current research demonstrates that LKB1 loss frequently co-occurs with KEAP1 mutation in KRAS-driven NSCLC. This co-mutation strengthens antioxidant defenses via the KEAP1/Nrf2 pathway, which scavenges radiotherapy-induced reactive oxygen species and further alters DNA damage repair efficiency (Sitthideatphaiboon et al., 2021). In addition, emerging evidence reveals that radiotherapy can reverse ferroptosis resistance in LKB1-loss tumors by activating the DNMT1-OSGIN1 axis, which subsequently triggers the STING-driven immune pathway. Notably, STING expression rises in a radiation dose-dependent manner. This crosstalk between metabolic regulation and anti-tumor immunity indicates that the DNMT1-OSGIN1 signaling cascade is a key mechanism by which radiotherapy overcomes ferroptosis tolerance in LKB1-loss tumors. The positive correlation between radiation dose and STING activation also highlights this axis as a tractable therapeutic target to improve clinical responses for LKB1-deficient lung malignancies (Zhang Y. P. et al., 2026).

Meanwhile, studies have indicated that the LKB1-AMPK-SLC7A11-GSH pathway modulates intracellular redox status. Mechanistically, LKB1 and AMPK deficiency reduces the transcription of the SLC7A11 gene, indicating a positive correlation between LKB1/AMPK activity and SLC7A11 expression (Rong et al., 2025). Although this study did not discuss the ferroptosis of lung cancer cells in the experiment, it clearly showed a relationship between LKB1-AMPK axis and SLC7A11-GSH-ROS axis, indirectly demonstrating that LKB1 deficiency in the absence of other factors may inhibit GSH production, thereby sensitizing cells to ferroptosis. In LKB1-loss cells, the massive synthesis of fatty acids due to persistent ACC activation will extremely deplete intracellular NADPH, resulting in the inability of glutathione reductase (GR) to reduce oxidized GSSG to GSH. This implies that when a large amount of ROS produced by radiotherapy attacks the biomembrane, LKB1-loss lung cancer cells tend to induce ferroptosis due to the blockage of GSH synthesis and malfunction of the antioxidant system (Li et al., 2020) (Figure 4).

LKB1 deletion exerts a paradoxical bidirectional regulatory effect on ferroptosis and radiosensitivity. LKB1 deletion itself is metabolically altered, which is an intrinsic property of the molecule. In the absence ofexogenous stressors, it can reduce the GSH level of cells and has the potential to promote ferroptosis. Lacking compensatory oncogenic mutations, normal somatic cells will exhibit ferroptosis sensitivity. Mechanistically, LKB1 deficiency suppresses AMPK activity, which universally reduces intracellular GSH levels in both tumor and normal cells by inhibiting SLC7A11-mediated cysteine uptake and inducing excessive fatty acid synthesis that depletes NADPH, thereby impairing antioxidant capacity and predisposing cells to ferroptosis. However, in clinically prevalent KRAS-driven and LKB1-loss NSCLC, tumor-specific compensatory mechanisms reverse this pro-ferroptotic phenotype. Concurrently, KEAP1 mutations activate the Nrf2 antioxidant pathway to compensate for GSH depletion and eliminate radiotherapy-induced ROS. Meanwhile, AMPK inactivation upregulates SCD1, which remodels cellular fatty acid composition by accumulating stable monounsaturated fatty acids and reducing peroxidation-prone PUFAs, ultimately blocking lipid peroxidation and ferroptosis. Abnormal activation of mTOR remodels metabolism and replenishes NADPH to maintain the GSH cycle (Lan et al., 2023). These synergistic tumor adaptive mechanisms counteract the inherent pro-ferroptotic metabolic defects caused by LKB1 loss, collectively conferring ferroptosis resistance and radioresistance on LKB1-deficient lung cancer cells. Nevertheless, this conjecture warrants further investigation.

KEAP1, a key negative regulator of Nrf2, modulates the radiosensitivity and ferroptosis of lung cancer cells primarily through Nrf2 and its downstream target molecules. Under physiological conditions, KEAP1 binds tightly to Nrf2 to mediate its ubiquitination and proteasomal degradation, keeping basal Nrf2 activity low. KEAP1 mutation disrupts this binding interaction, markedly increases Nrf2 protein stability, and promotes its nuclear translocation. Nuclear Nrf2 activates antioxidant response element (ARE)-dependent transcription, keeping cancer cells in a persistent antioxidant state. This potent antioxidant capacity eliminates radiotherapy-generated ROS and ferroptosis-related free radicals, ultimately blunting treatment sensitivity. Clinical data has verified that the KEAP1/Nrf2 axis is significantly correlated with a higher risk of local tumor recurrence, demonstrating that such genetic mutations contribute to unsatisfactory radiotherapy outcomes in lung cancer (Bourbonne et al., 2024). (Figure 4) KEAP1 mutations upregulate activity of the CoQ-FSP1 axis, which effectively blocks lipid peroxidation and suppresses ferroptosis, thereby reinforcing radioresistance in lung cancer cells. Targeted inhibition of FSP1 and Nrf2 has been explored as a therapeutic strategy for KEAP1-mutant NSCLC, with experimental outcomes supporting its efficacy (Kim et al., 2023; Tang et al., 2026). Due to the shifting of ferroptosis defense mechanisms, the FSP1-CoQ axis has emerged as a major pathway for radioresistance in KEAP1 mutant lung cancer. Metabolomics analysis showed that CoQ synthesis was upregulated and glutathione synthesis was downregulated in the acquired radioresistant cells. This explains why FSP1 knockdown is more effective than GPX4 knockdown in sensitizing cells to radiation therapy (Lin et al., 2024).

Epidermal growth factor receptor (EGFR) mutation is one of the most common mutations in NSCLC, mainly including exon 19 deletion (Del19) and exon 21 L858R point mutation. In addition, acquired resistance mutations (T790M) have emerged after first-generation or second-generation EGFR tyrosine kinase inhibitors (TKIs). Clinical analysis has shown that patients with EGFR mutation-positive NSCLC are generally more sensitive to radiotherapy. After exposure to different doses of ionizing radiation, the survival rate of EGFR-mutation cell lines is significantly lower than that of mutation-negative cell lines, indicating that this mutation may enhance radiosensitivity (Tanaka et al., 2023a). This may be related to the impaired DNA repair capacity caused by the mutation, DNA synthesis or mitotic cycle arrest was also observed in previous experiments, along with the occurrence of apoptotic phenomena (Das et al., 2006). The effect of different mutation sites on DNA repair capacity is certainly different. However, this difference is not observed *in vitro* experiments (Xie et al., 2018), but evidence in clinical practice. The researchers observed that after EGFR-TKIs and radiotherapy, the prognosis of patients with Del 19 mutation was significantly better than that of patients with L858R mutation (51.0hVS21.9h, P = 0.0013) (Liang S. et al., 2024). Considering the complex genetic mutation background in human body, the occurrence of this phenomenon may be caused by co-mutation. Of course, the role of the tumor microenvironment in the human body cannot be ignored. In current clinical practice, the drug selection of advanced lung cancer has been changed according to the gene mutation (EGFR mutation using TKI), but the radiotherapy plan, including the radiation dose, frequency, and radiation field, has not been adjusted according to the gene mutation status. Moving forward, personalized radiotherapeutic regimens tailored to EGFR genotypes warrant further exploration. For EGFR-positive patients, optimized dose modulation combined with adjuvant radiosensitizing agents may simultaneously boost anti-tumor efficacy and alleviate treatment-related toxicities.

KRAS mutation has a high incidence in lung cancer. However, studies on its relationship with radiosensitivity or ferroptosis are limited. One study has found that tumor cells harboring KRAS mutation are generally insensitive to radiotherapy, and the mechanism is closely related to the inhibition of ferroptosis. This may be attributed to the increased DNA damage repair ability and the upregulated Nrf2 expression caused by KRAS mutation, thereby enhancing cell survival rate (Yang et al., 2021). In patients with colorectal cancer (CRC), KRAS mutation is often accompanied by altered expression of genes related to ferroptosis inhibition, which is significantly associated with poor prognosis (Yan et al., 2023). Existing studies mainly focus on the effects of KRAS mutation on signaling pathways (Raf/MEK/ERK, PIK3/AKT), as well as its impacts on cell proliferation, survival and drug resistance, while relatively few studies have explored its influence on novel cell death modes. Some studies do explore ferroptosis, but the overall research accumulation is small, and there is no broad consensus at present.

In order to translate the above evidence based on gene mutation analysis into a clinically executable model, a four-step decision-making process was established using indicators that can be obtained by routine next-generation sequencing (NGS) and standard pharmacodynamic testing. First, perform comprehensive NGS genomic detection to characterize concurrent oncogenic variants, with priority assigned to four core driver genotypes: isolated TP53 mutation/deletion, isolated KEAP1/NFE2L2 loss or mutation, isolated STK11/LKB1 deletion, and compound co-mutation subsets carrying two or more of the above alterations. Owing to insufficient mechanistic data linking KRAS/EGFR variants to ferroptosis-radiation crosstalk, these two mutation subgroups are excluded from the current stratified therapeutic paradigm and require further preclinical validation before clinical incorporation. All accompanying secondary gene variants are documented to account for confounding metabolic background. Second, the ferroptosis defense pathway that becomes the rate-limiting link after radiotherapy is inferred. For example, TP53 mutant tumors use the System Xc^−^-GSH pathway as the rate-limiting node, and KEAP1/NFE2L2 mutant tumors shift their core defense pathway to the FSP1-CoQH_2_ pathway. In LKB1 deficient tumors, lipid remodeling, NADPH supply and mitochondrial electron transport together constitute the rate-limiting link of ferroptosis resistance. Third, drugs that target the corresponding rate-limiting node rather than broad-spectrum ferroptosis inducers were selected, as described in Table 2. Fourth, pharmacodynamic readouts (C11-BODIPY lipid ROS, 4-HNE adducts, PTGS2 expression, GSH/GSSG ratio, CoQH_2_/CoQ ratio, DLAT oligomers and so on) were used to verify the efficacy. At present, the existing literature suggests that when KEAP1/NFE2L2 mutation is combined with TP53 mutation or STK11/LKB1 mutation, the tumor exhibits a Nrf2-dominated antioxidant phenotype, preferentially blocking the FSP1-CoQ pathway, rather than interfering with the GSH pathway. Moreover, in KRAS-driven tumors with STK11/LKB1 deletion, glutamine compensatory metabolism becomes the rate-limiting step in antioxidant capacity, and glutaminase inhibition is the preferred adjuvant therapy.

**TABLE 2:** Geno-guided conception framework concerning patients stratification.

Driver genotype (frequency in NSCLC)	Dominant ferroptosis defense node after IR	Predicted phenotype susceptibility/radiosensitivity	Mechanistically matched intervention	Representative pharmacological or clinical evidence	Genotype-matched cuproptosis co-induction	Candidate pharmacodynamic readout	References
TP53 mutant or null (∼50%)	Loss of p53-mediated transcriptional repression of SLC7A11 System Xc^−^-GSH axis becomes dominant	Ferroptosis-tolerant and relatively radioresistant Radiosensitivity partially restorable by ferroptosis induction	Restoration of p53 pathway activity combined with System Xc^—^GSH-axis blockade. Ferroptosis inducer	p53 restoration after irradiation increases lipid peroxides and PTGS2 Increased cell rate after ferrostatin-1, confirmed in organoids. p53 activation sensitizes NSCLC to combination therapy	Low priority GSH depletion secondarily lowers cytosolic Cu ions buffering Co-induction is plausible but unsupported	Lipid ROS (C11-BODIPY) 4-HNE adducts PTGS2 mRNA GSH/GSSG ratio	(Lei et al., 2021; Choi et al., 2024)
KEAP1/NFE2L2 mutant (∼20%)	Nuclear Nrf2 activates ARE-dependent gene transcription KEAP1 Mutation activates FSP1-CoQH_2_	Most ferroptosis-resistant and poor radiotherapy outcomes	FSP1 inhibition as first choice, with NRF2 inhibition and/or USP7 inhibition GPX4 inhibition alone is predicted to be insufficient	FSP1 confers resistance in KEAP1-mutant NSCLC. FSP1 knockdown resensitizes more effectively than GPX4 knockdown in acquired radioresistance	Predicted cuproptosis-tolerant because Nrf2-driven SLC7A11/GSH also buffers Cu ions. Major antioxidant pathway, FSP1-CoQH_2_, is not directly associated with copper mortality	CoQH2/CoQ ratio FSP1 target engagement, NQO1/HMOX1 as NRF2 output, lipid ROS	(Koppula et al., 2022; Kim et al., 2023; Lin et al., 2024; Tang et al., 2026)
STK11/LKB1 deficient (∼15–20%,frequently co-mutant with KRAS and/or KEAP1)	AMPK hypoactivity leads to SCD1/MUFA mediated membrane protection and ACC-driven NADPH drain ETC complex I and LKB1-AMPK axis inactivation DNMT1-OSGIN1- STING axis activation Nrf2 compensation when KEAP1 is co-mutated	Bidirectional and context-dependent: clinically worse radiotherapy outcome when Nrf2 compensation is present Ferroptosis-prone when NADPH-dependent GSH regeneration fails	SCD1 inhibition and MUFA restriction plus glutaminase inhibition when KEAP1 is co-mutated Cuproptosis inducer Fractionation RT designed to activate the DNMT1- OSGIN1-STING axis	LKB1-AMPK inhibits ferroptosis via lipid metabolism SCD1 causes radioresistance. LKB1 predicts radiotherapy prognosis in stage III NSCLC. LKB1-mutant tumours develop KEAP1/Nrf2-dependent radioresistant phenotypes reversible by glutaminase inhibition IR reduces DNMT1 to reactivate silenced STING.	Highest-priority: glutamine-dependent TCA/OXPHOS metabolism intersects with cuproptosis. Combination of cuproptosis and radiotherapy is predicted to exert synergistic anti-tumour effects	MUFA/PUFA ratio NADPH/NADP (+) GSH/GSSG STING-dependent interferon signature DLAT oligomers	(Galan-Cobo et al., 2019; Li et al., 2020; Tsvetkov et al., 2022; Sen et al., 2023; Mao et al., 2024; Sitthideatphaiboon et al., 2024)
Composite co-mutant: KRAS + STK11/LKB1 (with KEAP1)	Supra-additive antioxidant compensation through Nrf2 activation and glutamine-dependent anaplerosis	Predicted to be the most ferroptosis-tolerant and radioresistant composite phenotype	Glutaminase inhibition plus FSP1 inhibition Cuproptosis co-induction as the alternative option	LKB1 and KEAP1/Nrf2 pathways cooperatively promote metabolic reprogramming with enhanced glutamine dependence. co-mutation frequency and clinical significance documented in KRAS-driven NSCLC.	Alternative option Cuproptosis interventions should be considered when there is no effect to avoid toxicity	Glutamine flux surrogates, GSH/GSSG CoQH2/CoQ, DLAT oligomers	(Galan-Cobo et al., 2019; Pons-Tostivint et al., 2021; Sitthideatphaiboon et al., 2021)

In summary, lung cancer features diverse molecular subtypes driven by distinct gene mutations, so radiosensitization strategies should be tailored to individual mutation backgrounds. It is insufficient to regulate IR-induced ferroptosis merely from the perspective of genetic alterations. The initiation and progression of ferroptosis rely on the coordinated function of multiple pathways, including iron metabolism, lipid metabolism, and redox homeostasis, and are further modulated by external factors such as tumor microenvironment and therapeutic interventions. As stable and intrinsic characteristics, gene mutations cannot rapidly adapt to intracellular environmental changes triggered by IR, thus failing to fully explain the complex regulatory network of ferroptosis. RNA, proteins and other factors can also partly explain this mechanism.

#### 3.2.2 Regulatory mechanisms of RNA

Upon IR exposure, tumor cells strengthen their antioxidant and cytoprotective defenses via multiple mechanisms to counteract ferroptosis and establish radioresistance. Radiosensitive and radioresistant tumor cells display distinct RNA expression profiles and epigenetic landscapes, with each subtype possessing unique transcriptional signatures. Following irradiation, sensitive cells undergo widespread transcriptomic alterations, showing transient activation of pro-apoptotic, pro-ferroptotic and repair-associated genes that eventually become depleted. In contrast, radioresistant cells develop durable adaptive phenotypes, preventing cell death through m6A modification and ceRNA regulatory networks, and suppressing ferroptosis, apoptosis and other cell death pathways. Lung cancer is shaped by smoking exposure, intricate genetic mutations and persistent oxidative stress, making it difficult to identify a universal biomarker for accurate radiotherapy response prediction. MicroRNAs (miRNAs) are a class of non-coding RNAs (ncRNAs) approximately 22 nt in length. They mediate mRNA degradation and translational repression by binding to the 3′UTR of target transcripts. Accumulating evidence demonstrates that IR profoundly remodels miRNA expression, distribution and biological function. Numerous studies have confirmed that miRNAs directly target core genes involved in ferroptosis, such as GPX4 and Nrf2, as well as key regulators of iron and lipid metabolism. By modulating these pathways, miRNAs govern the ferroptosis sensitivity of lung cancer cells and ultimately influence radiotherapy efficacy (Lv et al., 2026). Targeting miRNAs can also regulate the activity of CD8^+^T cells, providing a new strategy for immunotherapy combined with radiotherapy. Since the function of miRNAs is significantly dependent on the cellular environment and they can bind to different mRNAs to work under different pathophysiological conditions, their functions need to be verified by *in vitro* and *in vivo* models to clarify its biological effects. In addition, dual luciferase reporter assay is an efficient method to verify the direct interaction between miRNAs and their target genes and confirm the regulatory relationship (Wu et al., 2021).

As the most abundant RNA modification in eukaryotes, the regulatory core of m6A modification includes writers (METTL3/4, etc.), erasers (FTO, ALKBH5, etc.) and readers (IGF2BP1/2/3, YTHDF/YTHDC1/2, etc.). m6A modification dynamically regulates RNA splicing, translation, and stability. m6A modification mediated by methyltransferase (METTL3) plays a vital role in radioresistance (Xiang et al., 2017). For example, the levels of METTL3 and its mediated m6A modification are increased in NSCLC after carbon-ion irradiation (Xu X. et al., 2023).At the same time, studies have found that lncRNA CBR3-AS1 has a clear role in promoting radioresistance, and its expression is highly dependent on m6A modification (Hu et al., 2025). SLC7A11 is also a key target of m6A modification and is bi-directionally regulated by a variety of m6A reading proteins. YTHDC2 increases the instability of SLC7A11 mRNA and accelerates its degradation in a m6A-dependent manner (Ma et al., 2021).Some studies have suggested that the YTHDC2/SLC7A11/GSH pathway can induce ferroptosis (Zhang et al., 2025a). However, YTHDC1 stabilizes SLC7A11 transcription by recognizing m6A modification and ultimately enhances ferroptosis resistance (Xu Y. et al., 2022).The m6A-ferroptosis-radiotherapy axis is fully demonstrated in other diseases. It has been reported that m6A demethylase FTO enhances the radioresistance of nasopharyngeal carcinoma (NPC) cells by promoting OTUB1-mediated ferroptosis inhibition (Huang et al., 2023). There exists a clear divergence in research findings concerning the demethylase FTO. By contrast, in lung squamous cell carcinoma models, FTO modifies ACSL4 transcripts to elevate lipid activation levels and thereby potentiate radiotherapy-triggered ferroptosis. The same modifying enzyme exerts completely opposite functions across distinct pathological subtypes of lung cancer (Kuganesan et al., 2026). Current reviews rarely dissect m6A-mediated regulatory patterns according to pathological subtypes. Generalizing FTO merely as a target for radioresistance may readily introduce biases in subsequent translational investigations.

At present, the research on lncRNA and circRNA regulating the radiosensitivity of lung cancer through ferroptosis mechanism remains at an early exploratory phase. Existing reports mostly focus on ceRNA regulatory network. lncRNA H19 in NSCLC mediates the sensitivity of X-ray and carbon-ion irradiation through the lncRNA H19-miR-130a-3p-WNK3 signal axis through apoptosis. Specifically, H19 functions as a ceRNA by sponging miR-130a-3p upon H19 silencing (Zhao et al., 2021). Separately, in curcumin-triggered ferroptosis models of lung cancer cells, H19 sequesters miR-19b-3p to relieve post-transcriptional repression of FTH1. This process ultimately downregulates key ferroptosis-associated proteins including GPX4, Nrf2 and FTH1, supporting the functional involvement of H19 (Zhang R. et al., 2022). Future studies should focus on the combined role of H19 in regulating ferroptosis and radiosensitivity. Similarly, circPVT1 can adsorb miR-338-3p, upregulate GPX4 levels, and inhibit ferroptosis in the NSCLC model. When GPX4 activity is decreased by IR, promoting circPVT1 may partially improve the effect of radiotherapy (He et al., 2025). For radioresistant patients, RNA expression is the result of adaptive cell resistance. To this end, a therapeutic model that blocks RNA adaptation before radiotherapy can be developed in the future.

#### 3.2.3 Regulatory mechanisms of protein modifications

Protein post-translational modifications (PTMs) refer to chemical modifications after protein synthesis, including ubiquitination, phosphorylation, acetylation, SUMOylation, lactoylation and other forms. PTMs widely affect the sensitivity of cells to oxidative stress by regulating the functional state of ferroptosis core proteins. For example, the stability of GPX4 and SLC7A11 is precisely regulated by E3 ubiquitin ligases and deubiquitinases, and their level of ubiquitination directly determines cellular antioxidant capacity (Liu X. et al., 2026). These findings reveal the critical role of PTMs in the regulation of ferroptosis. Radiotherapy itself can induce changes in the spectrum of specific PTMs, such as reprogramming of ubiquitination, acetylation and phosphorylation, which in turn affect the function of ferroptosis-related proteins (Ju et al., 2025). Targeting key enzymes of PTMs can reduce ferroptosis threshold and achieve radiosensitization (Yan Y. X. et al., 2025). Hat1-mediated ACSL4 acetylation not only promotes tumor malignant progression but also enhances radiotherapy-induced ferroptosis in NPC models (Zhou et al., 2025).

Ubiquitin signaling exerts opposing effects on radiotherapy response, which can be categorized into two functionally distinct clusters, including radiosensitizing E3 ligases and radioresistance-associated E3 ligases/deubiquitinases. Sensitizing E3 ubiquitin ligases (FBXW7, TRIM36, FBXO22): These enzymes target DNA repair proteins and anti-ferroptotic factors such as GPX4 for proteasomal degradation upon irradiation, disrupting cellular antioxidant capacity and accelerating radiation-induced ferroptosis (De et al., 2021; Yu et al., 2022; Zhu et al., 2022). Their overexpression consistently lowers tumor survival rates post-radiation across multiple NSCLC cell lines. Resistance-driving ubiquitin machinery (CHIP, HDAC6, UBR5 and DUB family OTUB1, USP9X, OTUD4, UCHL3): CHIP and HDAC6 stabilize stemness and DNA damage repair proteins to counteract radiation toxicity. UBR5 activates the PI3K/AKT cascade to block ferroptosis signaling (Moses et al., 2020; Gu and Ge, 2021; Tan et al., 2023). Deubiquitinases further reinforce radioresistance via two conserved mechanisms: OTUB1 and USP9X stabilize CHK and other homologous recombination proteins to eliminate radiation-induced DNA lesions, while OTUD4/UCHL3 maintain high levels of SLC7A11 and GPX4 to sustain GSH-dependent antioxidant defense (Jie et al., 2021; Wang J. et al., 2024; Wu et al., 2019; Xu L. M. et al., 2022). Current studies only characterize individual ubiquitin enzymes in isolated cell systems, lacking head-to-head comparisons of their substrate networks across identical irradiation doses and mutant backgrounds. No research has clarified whether combined inhibition of multiple resistance-related DUBs produces synergistic radiosensitization, which hinders the development of ubiquitin-targeted combinatorial therapies.

At present, the radiosensitization strategy is no longer limited to GPX4, and the importance of FSP1 is also being taken seriously. In addition, the transcription-epigenetic-PTM axis also provides a broad decision space for combinatorial targeting strategies. Single-target interventions have been questioned accordingly.

### 3.3 Regulation of lipid metabolism

Different from the intracellular regulatory sites of antioxidant defense pathways, lipid metabolism modulates the radiosensitivity of lung cancer at the biological membrane level.

The peroxidation of PUFAs is the core stage of ferroptosis, and also a key link determining radiotherapy efficacy. Specifically, the content and proportion of PUFAs in biomembranes, as well as their integration status in membrane phospholipids, are critical determinants of the ferroptosis sensitivity in lung cancer cells. For example, the lipid profile of lung cancer cells is significantly changed after radiotherapy. The level of sterol lipids is increased, while glycerolipids such as triglycerides are significantly decreased, suggesting impaired lipid storage function and enhanced lipid droplet mobilization. In addition, radiotherapy can also cause changes in the unsaturation of glycerophospholipid family, which creates favorable conditions for ferroptosis (He et al., 2024). As the rate-limiting enzyme responsible for long-chain PUFA activation, ACSL4 remodels tumor lipid composition to increase membrane fluidity and facilitate lipid peroxidation, consequently lowering the threshold for ferroptosis execution (Wang et al., 2025c). In contrast, in radioresistant lung cancer cell lines, oleic acid (OA) and palmitoleic acid (POA) levels are significantly increased, reducing PUFA-phospholipid accumulation via ACSL3, reducing lipid peroxidation levels, inhibiting ferroptosis and leading to radiation resistance. Knockdown of ACSL3 can reverse this effect and enhance the antitumor effect of radiotherapy combined with ferroptosis inducers (Cao et al., 2025). Furthermore, epithelial-mesenchymal transition (EMT) can also alter lipid metabolism and regulate PUFAs content in cell membranes, which in turn affects membrane rigidity and lipid peroxidation. This also serves as one of the important factors influencing ferroptosis (Guo W. et al., 2025).

Cholesterol is an important component of cell membrane. It modulates membrane fluidity and facilitates lipid-raft assembly, consequently influencing the propagation of lipid peroxidation chain reactions (Zhao X. et al., 2023). In most biological contexts, cholesterol confers resistance to ferroptosis. However, certain cholesterol metabolites can disrupt cellular metabolism and promote ferroptosis. Increased cholesterol synthesis contributes to radioresistance. For instance, SCARB1 modulates cholesterol turnover and elevates cellular CoQ abundance to suppress lipid peroxidation and ferroptosis, ultimately driving radioresistance in tumor cells. Therapeutic targeting of this pathway is capable of reversing the resistant phenotype in SCARB1-high tumors (Mao et al., 2025). In addition, in the colorectal cancer model, radiotherapy can rapidly upregulate the expression of 3-hydroxy-3-methylglutaryl-CoA reductase (HMGCR), promote cholesterol synthesis and inhibit the cGAS-STING pathway, thereby impairing the radiosensitivity. Because this pathway (cGAS-STING) is conserved in a variety of solid tumors, it provides a relatively reliable reference for lung cancer research (Bao et al., 2024; Joshi and Izgu, 2026).In addition, targeted intervention of cholesterol metabolism has been verified to be feasible in head and neck cancer research (Radha et al., 2023).

Lipid droplets are the major storage form of neutral lipids, and their accumulation may buffer oxidative stress. In various cancer types, surviving cells after radiotherapy accumulate abundant lipid droplets, and lipid droplet abundance is positively correlated with FTH1expression. As a core component of ferritin, FTH1 is essential for maintaining iron homeostasis and inhibiting lipid peroxidation (Tirinato et al., 2021). It is therefore hypothesized that lipid droplets are functionally linked to, and may crosstalk with, ferroptosis pathways to mediate radioresistance. Lipid droplets accumulate significantly in radioresistant lung cancer cells, and photodynamic therapy (PDT) targeting lipid droplets can induce lipid peroxidation and ferroptosis, destroy DNA damage repair ability, and thus enhancing radiosensitivity (Xu S. et al., 2026). Lipid droplets accumulation is observed not only in lung cancer cells, but also in breast cancer cells and glioma cells, indicating a pan-cancer mechanism. Targeted therapy for such phenomena may represent a promising direction for future investigation (Tirinato et al., 2021). Similarly, IL-17D activates PPARγ-dependent lipid metabolic reprogramming, reduces intracellular lipid peroxidation, and induces ferroptosis resistance. Meanwhile, it promotes lipid droplet accumulation and ATP production, whereas PPARγ inhibition can reverse this phenotype (Du et al., 2026). Low-dose radiotherapy (LDR) studies have found that lipid droplet synthesis via FABP (fatty acid-binding protein) in tumor cells is a key factor in LDR-induced cytotoxicity and immune stimulation. Targeting this pathway can strengthen the therapeutic effect of radiotherapy via ferroptosis (Zhang Q. et al., 2023). Inhibition of lipid droplet biogenesis and metabolism represents an emerging therapeutic strategy to augment the radiosensitivity of malignant tumors. At present, the main limitation lies in the lack of clinical evidence. In addition, how to specifically target lipid droplet metabolism in tumor tissues while preserving normal tissue function remains a critical challenge for clinical translation.

Lipid metabolic reprogramming determines membrane peroxidation susceptibility, and lipid species can be categorized into two opposing functional groups that exert inverse effects on radiation-induced ferroptosis.

ACSL4 drives PUFAs activation and phospholipid incorporation, the essential substrate for lipid chain reactions. Radiotherapy consistently upregulates ACSL4 in radiosensitive tumor clones, while pharmacological ACSL4 activation effectively amplifies lipid ROS. ACSL3-mediated MUFAs synthesis, cholesterol-lipid raft assembly, and lipid droplet storage all mitigate peroxidation chain propagation. Radioresistant cells universally upregulate ACSL3 and SCD1 to replace PUFAs with stable MUFAs. Accumulated lipid droplets further sequester toxic oxidized lipids as a self-protective buffer.

Current literature separately reports individual lipid enzymes without unified metabolic network analysis. Few studies quantitatively compare the PUFAs/MUFAs ratio shift before and after irradiation. There remains no consensus on whether lipid droplets function as temporary protection or long-term resistance drivers in clinical tumors.

## 4 Crosstalk of ferroptosis and cuproptosis

Traditional radiotherapy alone has limited effect on inducing tumor cell killing, and it is still difficult to overcome the intrinsic or acquired tumor resistance to radiotherapy by using radiotherapy, chemotherapy and targeted drugs alone. The core idea of this study is to stratifying lung cancer driver gene mutations and targeting ferroptosis to achieve individualized sensitization. As a programmed cell death pathway that can cooperate with ferroptosis, cuproptosis can differentially affect the killing effect of radiotherapy according to different gene background, providing a complementary strategy for genotype-directed comprehensive treatment of lung cancer. Although apoptosis has long been regarded as the primary form of cell death induced by radiotherapy, increasing evidence has shown that ferroptosis and cuproptosis can effectively eliminate tumor cells (Xu W. et al., 2023). Cuproptosis is mainly triggered by copper ions overload, which causes abnormal accumulation of mitochondrial lipoylated proteins and dysfunction of iron-sulfur cluster proteins, ultimately leading to mitochondrial inactivation. It plays an independent role distinct from ferroptosis and apoptosis (Lei et al., 2025). This process is dependent on copper transporter (CTR1)-mediated copper influx and the regulation of key enzymes such as FDX1/LIAS. Several studies have confirmed the synergistic antitumor effects of ferroptosis and cuproptosis in various tumor models (Yan S. et al., 2025; Zhang et al., 2025b). Together, these three mechanisms illustrate the synergistic effect between ferroptosis and cuproptosis, which lays a theoretical foundation for the synergistic induction of cell death under radiotherapy. Furthermore, given the differences in sensitivity of tumors of different genotypes to the induction of cuproptosis, this synergistic effect may also be affected by mutations in genes such as p53, KEAP1, LKB1, EGFR, and KRAS. Therefore, it is essential to distinguish gene-induced differences in cuproptosis susceptibility for accurate gene stratification for radiotherapy.

### 4.1 Coupling effects of iron-copper ion metabolism

Both ferroptosis and cuproptosis depend on the abnormal accumulation of metal ions, and there is a significant coupling effect between iron and copper in intracellular metabolic pathways. Ferroptosis is driven by intracellular iron overload, which promotes the generation of ROS, leading to lipid peroxidation and biomembrane instability. Cuproptosis is triggered by the increase of copper ion concentration, which leads to the accumulation of lipoylated tricarboxylic acid cycle proteins and the loss of iron-sulfur cluster proteins in mitochondria. Studies have shown that iron and copper share key nodes in the regulation of redox homeostasis, such as glutathione system. GSH is not only an essential reducing agent for GPX4 to maintain ferroptosis inhibition, but also an important molecule for copper chelation and detoxification. Many studies have also used GSH depletion as a way to enhance cuproptosis (Gao et al., 2025; Zhang et al., 2025b). In addition, changes in the expression of copper transporters such as ATP7B not only affect copper homeostasis, but also may indirectly interfere with the function of iron metabolic-related proteins such as ferritin (FTH1) (Zhi et al., 2026). This evidence suggests that iron-copper metabolism does not operate independently, but rather forms a coupled network at multiple levels that together determine the sensitivity of cells to radiotherapy-induced death signals.

### 4.2 Pivotal role of mitochondrial damage

Mitochondria serve as a common key execution site for both ferroptosis and cuproptosis, and mitochondrial dysfunction exerts a cascade amplification effect in the synergistic interaction of the 2 cell death modes. Cuproptosis is strongly dependent on the activity of the mitochondrial respiratory chain. Copper ions bind to mitochondrial lipoylated proteins such as DLAT, trigger their oligomerization, and disrupt the tricarboxylic acid cycle (Xu L. et al., 2026). Traditionally, ferroptosis was considered to occur mainly in the plasma membrane. However, recent studies have shown that mitochondrial iron accumulation, decreased membrane potential and dysfunction of respiratory chain complexes are also important hallmarks of ferroptosis. In NSCLC, PHKG2 overexpression promotes ferritinophagy, increases mitochondrial iron load, and aggravates mitochondrial dysfunction, thereby enhancing radiation-induced ferroptosis (Han et al., 2024). Studies have shown that radiotherapy can upregulate the copper transporter1 (CTR1) and reduce the level of mitochondrial glutathione, leading to the accumulation of mitochondrial copper, and finally trigger cuproptosis (Lei et al., 2025). More importantly, the co-accumulation of Fe and Cu can synergistically disrupt the assembly of mitochondrial Fe-S cluster, which is mediated by proteins such as ISCA2, and its impairment will further weaken the function of, ETC and amplify oxidative stress (Luan et al., 2025). Nanoplatforms that target iron and copper ions to mitochondria significantly enhance ROS production and downregulate protective factors such as FDX1 and HSP70, achieving integrated activation of multi-pathway cell death signals (Liu et al., 2025). In addition, they interact with each other in the mitochondrial space. For example, ferroptosis leads to the depletion of heat shock protein 70(HSP70) and the decrease of ATP, which ultimately improves the sensitivity to cuproptosis (Liu M. et al., 2026). These evidences suggest that mitochondria in the context of radiotherapy is absolutely the site of occurrence of both modes of death, which also provides directions for more targeted therapy.

### 4.3 Thiol metabolic network

Thiol metabolism, especially GSH and its related enzyme system, constitutes a significant node of the cross-regulation of cuproptosis and ferroptosis. GSH is not only an essential cofactor for GPX4 to exert antioxidant functions, but also an important intracellular copper chelator involved in maintaining copper homeostasis. In cuproptosis, the reduction of mitochondrial GSH weakens the chelating ability to copper ions, resulting in the accumulation of free Cu^2+^ which binds to lipoacylated proteins and trigger proteotoxic stress (Lei et al., 2025). However, in ferroptosis, GSH depletion directly leads to the inactivation of GPX4 and the inability to effectively remove lipid peroxides, thus promoting the ferroptosis progress. Several studies have confirmed that targeting thiol metabolism activates both modes of death simultaneously (Wan et al., 2025). For example, CMSP nanoassembly inhibits System Xc- with Sorafenib and blocks cystine uptake, thus causing GSH biosynthesis disorder and releasing copper ions to induce the aggregation of lipoacylated proteins to achieve the synergy of dual pathways (Zhang M. et al., 2026).Low-dose radiotherapy combined with copper induction strategy also overcomes radiation resistance through continuous depletion of GSH and supply of copper ions (Shao et al., 2024). Therefore, the thiol metabolic pathway, which tightly couples copper homeostasis, lipid peroxidation, and mitochondrial function through GSH, a central molecule, is an ideal intervention target for the synergistic induction of dual programmed death.

### 4.4 Effect of gene on cuproptosis

The cuproptosis sensitivity is determined by redox buffer capacity, copper transport system and mitochondrial metabolism (Jin et al., 2026). Common genetic mutations, as well as some specific mutations, can reshape the sensitivity of cells to cuproptosis. However, no study has specifically explored the association between gene mutations, cuproptosis and radiosensitivity at present.

#### 4.4.1 P53 gene mutations

P53 gene exerts a regulatory effect on the sensitivity to cuproptosis. The maintenance or restoration of WT-p53 function can enhance the sensitivity of cells to cuproptosis, while mutant p53 may impair the sensitivity. The specific mechanism is closely related to metabolic reprogramming. As a key transcription factor, p53 can affect cuproptosis by regulating metabolic pathways. It was shown that returning mutant p53 to a WT conformation, such as treatment with APR-246, upregulated TP53-induced regulator of glycolysis and apoptosis (TIGAR) and glutaminase 2 (GLS2), inhibited glycolysis and enhanced TCA cycle flux. Since cuproptosis depends on the binding of copper ions to fatty acylated proteins in the TCA cycle, enhanced TCA cycle activity makes cells more susceptible to triggering cuproptosis, thereby sensitizing the tumor cell response to cuproptosis (Ma et al., 2026). In addition, the nanomaterial strategy successfully achieved self-sensitized cuproptosis by upregulating the expression of p53, which further verified that the increase of p53 level could enhance the effect of cuproptosis (Wang et al., 2025d). Overall, p53 is an important molecular switch that determines the sensitivity of cells to copper death by regulating TCA cycle metabolic flow and mitochondrial function. Its functional status (WT/mutant type) directly affects the efficiency of cuproptosis induction. Therefore, WT p53 enhances the efficiency of the tricarboxylic acid cycle, thereby enhancing the sensitivity of the cell response to copper death. Thus, conventional therapy may be the right choice for patients with p53 deficiency.

#### 4.4.2 KEAP1 gene mutation

The effect of KEAP1/Nfr2 pathway mutation on the sensitivity of cells to cuproptosis is mainly reflected in the reduction of the sensitivity of cells to cuproptosis. This pathway has a great influence on the intracellular redox state. Inactivation or mutation of KEAP1 or NFE2L2 leads to enhanced NRF2 protein stability and nuclear translocation, which continuously activates antioxidant response element (ARE) -driven gene expression, remodels cell metabolism and redox homeostasis, and thereby attenuates cuproptosis-induced cell death (Jin et al., 2026). Nfr2 activation can upregulate GSH synthesis related genes (such as GCLC, GCLM) and other antioxidant molecules, enhance the ability of cells to remove ROS and maintain copper homeostasis, and indirectly inhibit the aggregation of fatty acyl proteins and the damage of Fe-S cluster proteins caused by copper ion overload (Li et al., 2026). Notably, KEAP1-NFE2L2-CUL3 mutants exhibited disulfide stress and uncontrolled ROS in certain cancer types, such as endometrial cancer, suggesting that tissue background may influence the phenotype (Namani et al., 2024). But the overall trend still points to an inhibitory effect of pathway activation on cuproptosis.

#### 4.4.3 LKB1 gene mutations

The occurrence of cuproptosis is strictly dependent on TCA cycle activity and mitochondrial respiratory function. When cell metabolism shifts from glycolysis to dependence on TCA cycle and oxidative phosphorylation, the sensitivity to cuproptosis increases significantly (Xing T. et al., 2023). It is speculated that tumor subtypes dependent on TCA and oxidative phosphorylation are more sensitive to cuproptosis. Enhanced metabolism of glutamine, a key complement substrate of the TCA cycle (providing α-ketoglutarate), reinforces the dependence of cells on the TCA cycle and oxidative phosphorylation. Lung cancer cells with STK11/LKB1 deletion and KEAP1 inactivation undergo glutamine metabolic reprogram, with significant activation of the glutamine catabolization and complement pathway. Glutamine-derived α-ketoglutaric acid is highly dependent on glutamine-derived α-ketoglutaric acid to maintain the metabolic flow of the tricarboxylic acid cycle and mitochondrial oxidative phosphorylation, and glutamine products to synthesize GSH. Notably, LKB1 deficiency alone upregulated glycolysis, which was not dependent on oxidative phosphorylation. Only LKB1/KEAP1 double co-mutation was dependent on glutamine complementation to support TCA and mitochondrial oxidative phosphorylation (Faubert et al., 2014; Galan-Cobo et al., 2019). Therefore, STK11/LKB1 null tumors, especially those with KEAP1 co-mutation, are more prone to cuproptosis.

At present, there are few studies on the sensitivity of KRAS and EGFR mutations to copper death. The copper death pathway consists of 12 key genes whose expression and mutation directly affect the sensitivity of cells to copper death. However, some studies have clear conclusions or reasonable extrapolations concerning other genes. See Table 3.

**TABLE 3:** Fluence of other genes concerning cuproptosis sensitivity.

Genes	Dieases/Model	Regulation/Mechanism	References
SF3B1 mutation (K700E)	Acute myeloid leukemia (AML)	Up/SF3B1 mutation leads to abnormal splicing and downregulation of mitochondrial iron-sulfur cluster transporter ABCB7, resulting in iron-sulfur cluster defects	(Moison et al., 2024)
IDH1 mutation (R132H)	Acute myeloid leukemia (AML)	Up/Mutations perturb fat metabolism in tumor cells, making them more susceptible to interference by cuproptosis	(Zhang et al., 2026b)
IDH1 wild type (MYC amplification)	Medulloblastoma Group 3	Up/IDH1 gene promotes the high expression of MYC, and MYC transcription upregulates DLAT	(Dang et al., 2025)
12 Genes	bioinformatics analysis	FDX1, LIAS, LIPT1, DLD, DLAT, PDHA1 and PDHB, these genes contribute to the occurrence of cuproptosis MTF1, GLS, and CDKN2A, genes that are able to resist or inhibit the cuproptosis ATP7B and SLC31A1 are copper transporters. They are responsible for regulating intracellular copper ion homeostasis, thereby affecting cuproptosis	(Liu, 2022)
MT2A	mouse pulpitis model with MTII knockdown	Up/In related cell models, MT2A knockdown enhanced cuproptosis sensitivity, as shown by copper overload, increased cell death, and increased expression of DLAT	(Zhong et al., 2026)

In conclusion, cuproptosis can reverse ferroptosis resistance through multiple mechanisms (coinduction of coptoptosis and ferroptosis is also a way to reverse ferroptosis resistance). It mainly includes the ways of depleting iron metabolism, depleting GSH and aggravating mitochondrial damage.

Many studies have suggested that synchronous activation of ferroptosis and cuproptosis can induce immunogenic cell death (ICD) (Tang et al., 2025; Zhong et al., 2025), and cuproptosis induced by low-dose X-ray combined with nanomaterials can also reverse the immunosuppressive state of TME (Jiang et al., 2024). Therefore, a combination regimen combining cuproptosis induction, radiotherapy and immunotherapy is expected to maximize the therapeutic effect of lung cancer.

The current treatment strategy of combined cuproptosis has great potential in overcoming radioresistance. However, most of these studies are in the preclinical stage. The major challenge is how to avoid normal tissue toxicity, and there is a lack of efficacy markers. Given the relatively high systemic toxicity of free copper ions, nano-delivery systems hold greater promise for clinical translation. Drugs are attached to nanocrystals by radiosensitive chemical bonds, which are severed upon exposure to ionizing radiation, leading to local drug release and ROS production (Jiang R. et al., 2025). This design not only enhances the anti-tumor efficacy of radiotherapy, but also achieves the control of drug toxicity. Therefore, it is reasonable to hypothesize that future incorporation of cuproptosis and ferroptosis mechanisms into such nanomaterials could improve the efficacy of radiotherapy while reducing Cu toxicity.

It is worth emphasizing that tumor driver genes such as TP53, KEAP1, STK11/LKB1, IDH1 and MYC can separately affect the sensitivity of cells to cuproptosis and ferroptosis. The study of these and their combination is a promising but also challenging direction. In the future, the concept of genotype stratification can be integrated into the design of radiosensitization nanoplatforms to accurately activate the dual cell death pathway in tumors with specific mutation subtypes. In summary, individualized radiotherapy based on driver genotyping combined with radiation-responsive nanomedicine targeting ferroptosis and cuproptosis is expected to break the pattern of radioresistance of NSCLC and improve the clinical prognosis of patients.

## 5 Future directions

Based on current research and to further clarify the relationship between radiotherapy sensitivity and ferroptosis in lung cancer, future studies should focus on feasible basic research and clinical translation that conform to the real-world clinical context, which also reflects the current research bottlenecks.

In terms of basic research, existing studies generally lack unified and reproducible lung cancer cell lines and animal models with defined mutations. Lung cancer exhibits profound intratumoral heterogeneity, and concurrent co-mutations are highly prevalent in clinical practice. Consequently, single-mutation models fail to faithfully recapitulate real-world tumor biology, leading to fragmented and inconsistent mechanistic validation. Targeted therapy against a single genetic alteration is unlikely to achieve durable efficacy in co-mutant tumors, posing substantial challenges for clinical translation. Future investigations should prioritize high-frequency co-mutation subtypes, such as KRAS-LKB1 and KEAP1-LKB1, and employ patient-derived organoids (PDOs) and patient-derived xenografts (PDXs) to systematically dissect the integrated regulatory roles of DNA alterations, RNA dysregulation, and post-translational modifications (PTMs).

Nanomaterials and inducers differ significantly between *in vitro* and *in vivo* models. In the future, unified criteria for efficacy evaluation and safety are needed. Unified standards can compare data from different experiments and accelerate clinical translation.

The disturbance of emerging environmental factors has not been taken seriously. Due to changes in lifestyle, people have been increasingly exposed to chemicals such as microplastics, which have been shown to affect radiosensitivity through ferroptosis pathway (Zhou et al., 2026). Future research needs to conduct targeted studies and prevention on the impact of microplastics, and it is imperative to assess the comprehensive impact of microplastics on the efficacy of radiotherapy.

## 6 Conclusion

Radiotherapy resistance severely restricts the therapeutic effect of lung cancer. Ferroptosis is an important mechanism through which ionizing radiation kills tumor cells. This review systematically summarizes multi-layered regulatory networks linking ferroptosis to radioresistance, including gene mutations, RNA epigenetics, protein modifications and lipid metabolism. We classified radiosensitivity based on core NSCLC driver genotypes (TP53, KEAP1, LKB1 and combined mutations) and proposed genotype-specific targeted ferroptosis strategies to reverse radiation tolerance.

Ferroptosis and cuproptosis share mitochondrial and thiol metabolic hubs, and their combined induction exerts synergistic anti-tumor effects, especially in LKB1/KEAP1 co-mutant radioresistant lung cancers. Radiation-responsive nanomedicines offer a feasible translational tool to co-activate the 2 cell death pathways. In summary, genotype-guided ferroptosis targeting combined with cuproptosis induction represents a promising approach to overcome lung cancer radioresistance and optimize clinical radiotherapy outcomes.

## Glossary

4DCT: Four-Dimensional Computed Tomography
ACC1: Acetyl-CoA Carboxylase 1
ACC2: Acetyl-CoA Carboxylase 2
ACSL3: Acyl-CoA Synthetase Long-Chain Family Member 3
ACSL4: Acyl-CoA Synthetase Long-Chain Family Member 4
ALOX15: Arachidonate 15-Lipoxygenase
ALOXs: Arachidonate Lipoxygenases
AMPK: AMP-Activated Protein Kinase
ARE: Antioxidant Response Element
ATP7B: ATPase Copper Transporting β
BH4: Tetrahydrobiopterin
CAT: Catalase
CMSP: CuMOF-Sorafenib Nanoassembly
CoQ: Coenzyme Q
CoQH2: Dihydrocoenzyme Q
CPT-1: Carnitine Palmitoyltransferase 1
CRC: Colorectal Cancer
CTR1: Copper Transporter 1
Del19: Exon 19 Deletion
DFO: Deferoxamine
DHODH: Dihydroorotate Dehydrogenase
DLAT: Dihydrolipoamide S-Acetyltransferase
DMT1: Divalent Metal Transporter 1
DNMT1: DNA Methyltransferase 1
DNSP: Downshifting Nanoparticle
DSB: Double-Strand Break
EGFR: Epidermal Growth Factor Receptor
EMT: Epithelial–Mesenchymal Transition
ESCC: Esophageal Squamous Cell Carcinoma
ETC: Electron Transport Chain
FABP: Fatty Acid-Binding Protein
FBLN5: Fibulin 5
FBXO22: F-Box Protein 22
FBXW7: F-Box and WD Repeat Domain Containing 7
FDX1: Ferredoxin 1
FPN1: Ferroportin 1
FSP1: Ferroptosis Suppressor Protein 1
FTH1: Ferritin Heavy Chain 1
FTO: Fat Mass and Obesity-Associated Protein
GCH1: GTP Cyclohydrolase 1
GLS: Glutaminase
GPX1: Glutathione Peroxidase 1
GPX4: Glutathione Peroxidase 4
GR: Glutathione Reductase
GSH: Glutathione
GSTP1: Glutathione S-Transferase P1
Hat1: Histone Acetyltransferase 1
HCC: Hepatocellular Carcinoma
HDAC6: Histone Deacetylase 6
HMGCR: 3-Hydroxy-3-Methylglutaryl-CoA Reductase
HNRNPA2B1: Heterogeneous Nuclear Ribonucleoprotein A2/B1
HO-1: Heme Oxygenase 1
HSP70: Heat Shock Protein 70
ICD: Immunogenic Cell Death
ICIs: Immune Checkpoint Inhibitors
IGF2BP1/2/3: Insulin-Like Growth Factor 2 mRNA-Binding Protein 1/2/3
IR: Ionizing Radiation
IRP1: Iron Regulatory Protein 1
IRP2 I: ron Regulatory Protein 2
ISCA2: Iron-Sulfur Cluster Assembly 2
KEAP1: Kelch-Like ECH-Associated Protein 1
KRAS: Kirsten Rat Sarcoma Viral Oncogene Homolog
LDR: Low-Dose Radiotherapy
LIAS: Lipoic Acid Synthetase
LIP: Labile Iron Pool
LKB1: Liver Kinase B1
LOXs: Lipoxygenases
LPCAT3: Lysophosphatidylcholine Acyltransferase 3
MAFF: MAF BZIP Transcription Factor F
MAMs: Mitochondria-Associated Endoplasmic Reticulum Membranes
MC I: Mitochondrial Complex I
MCL1: Myeloid Cell Leukemia 1
METTL3: Methyltransferase-Like 3
MFRN1: Mitoferrin-1
MFRN2: Mitoferrin-2
miRNAs: MicroRNAs
MtFer: Mitochondrial Ferritin
MUFA: Monounsaturated Fatty Acids
NCOA4: Nuclear Receptor Coactivator 4
ncRNAs: Non-Coding RNAs
NF-κB: Nuclear Factor Kappa B
NPC: Nasopharyngeal Carcinoma
Nrf2: Nuclear Factor Erythroid 2-Related Factor 2
NSCLC: Non-Small Cell Lung Cancer
NTBI: Non-Transferrin-Bound Iron
OA: Oleic Acid
OPA1: Optic Atrophy 1
OTUB1: OTU Deubiquitinase, Ubiquitin Aldehyde Binding 1
OTUD4: OTU Deubiquitinase 4
PBX1: PBX Homeobox 1
PD-1: Programmed Cell Death Protein 1
PDT: Photodynamic Therapy
PHKG2: Phosphorylase Kinase Catalytic Subunit Gamma 2
POA: Palmitoleic Acid
PPARγ: Peroxisome Proliferator-Activated Receptor γ
PRDX6: Peroxiredoxin 6
PS-MPs: Polystyrene Microplastics
PTEN: Phosphatase and Tensin Homolog
PTGS2: Prostaglandin-Endoperoxide Synthase 2
PTMs: Post-Translational Modifications
PUFAs: Polyunsaturated Fatty Acids
RBMS1: RNA Binding Motif Single Strand Interacting Protein 1
ROS: Reactive Oxygen Species
RSL3: RAS-Selective Lethal 3
RT: Radiotherapy
SBRT: Stereotactic Body Radiation Therapy
SCARB1: Scavenger Receptor Class B Member 1
SCD1: Stearoyl-CoA Desaturase 1
SCLC: Small Cell Lung Cancer
SELENOK: Selenoprotein K
SLC7A11: Solute Carrier Family 7 Member 11
SOAT1: Sterol O-Acyltransferase 1
SOD2: Superoxide Dismutase 2
STAT3: Signal Transducer and Activator of Transcription 3
STEAP: Six-Transmembrane Epithelial Antigen of Prostate
STK11: Serine/Threonine Kinase 11 (LKB1)
**System Xc** ^**-**^: Cystine/Glutamate Antiporter System
TCA: Tricarboxylic Acid Cycle
TfR1: Transferrin Receptor 1
TKIs: Tyrosine Kinase Inhibitors
TME: Tumor Microenvironment
TRIM36: Tripartite Motif Containing 36
TRPA1: Transient Receptor Potential Ankyrin 1
UBR5: Ubiquitin Protein Ligase E3 Component N-Recognin 5
UCHL3: Ubiquitin C-Terminal Hydrolase L3
USP9X: Ubiquitin Specific Peptidase 9 X-Linked
WNK3: WNK Lysine Deficient Protein Kinase 3
YTHDC1: YTH Domain Containing 1
YTHDC2: YTH Domain Containing 2
YTHDF: YTH N6-Methyladenosine RNA Binding Protein F
ZIP14: Zrt- and Irt-Like Protein 14 (SLC39A8)
ZIP8: Zrt- and Irt-Like Protein 8 (SLC39A14)
4-HNE: 4-Hydroxynonens
